# Mathematical modeling identifies Lck as a potential mediator for PD-1 induced inhibition of early TCR signaling

**DOI:** 10.1371/journal.pone.0206232

**Published:** 2018-10-24

**Authors:** Theinmozhi Arulraj, Debashis Barik

**Affiliations:** 1 Centre for Systems Biology, School of Life Sciences, University of Hyderabad, Central University P.O., Hyderabad, Telangana, India; 2 School of Chemistry, University of Hyderabad, Central University P.O., Hyderabad, Telangana, India; University of Louisville, UNITED STATES

## Abstract

Programmed cell death-1 (PD-1) is an inhibitory immune checkpoint receptor that negatively regulates the functioning of T cell. Although the direct targets of PD-1 were not identified, its inhibitory action on the TCR signaling pathway was known much earlier. Recent experiments suggest that the PD-1 inhibits the TCR and CD28 signaling pathways at a very early stage ─ at the level of phosphorylation of the cytoplasmic domain of TCR and CD28 receptors. Here, we develop a mathematical model to investigate the influence of inhibitory effect of PD-1 on the activation of early TCR and CD28 signaling molecules. Proposed model recaptures several quantitative experimental observations of PD-1 mediated inhibition. Model simulations show that PD-1 imposes a net inhibitory effect on the Lck kinase. Further, the inhibitory effect of PD-1 on the activation of TCR signaling molecules such as Zap70 and SLP76 is significantly enhanced by the PD-1 mediated inhibition of Lck. These results suggest a critical role for Lck as a mediator for PD-1 induced inhibition of TCR signaling network. Multi parametric sensitivity analysis explores the effect of parameter uncertainty on model simulations.

## Introduction

Activation and subsequent proliferation of T cell are crucial events preceding pathogen clearance. However, proper functioning of the immune system also relies on the ability of T cells to promote self-tolerance. Hence, these processes are tightly controlled at multiple levels by regulatory mechanisms[1]. T cells have co-stimulatory and co-inhibitory receptors that coordinate to modulate its response[2]. TCR (T cell receptor) activation is primarily responsible for the activation of effector functions of T cells and its full activation needs co-stimulation by CD28 (Cluster of Differentiation 28) receptor [3, 4]. Induction of TCR and CD28 signaling pathways result in T cell proliferation, increased glucose uptake and production of cytokines [5]. On the other hand, inhibitory receptors CTLA-4 (Cytotoxic T-lymphocyte-associated antigen 4) and PD-1 (Programmed Cell Death-1) negatively regulate the T cell response. Activation of PD-1 receptor has been shown to negatively affect several processes upregulated by the TCR and its associated co-stimulatory signaling pathways[6, 7].Knockouts of the genes encoding these inhibitory receptors have produced autoimmune phenotypes in the animal models suggesting their role in preventing autoimmune diseases [8–10].

The finding that cancer cells can be recognized and destroyed by the immune system, has established the field of cancer immunology and the interaction between cancer cells and immune system is being studied extensively [11, 12]. Cancer cells are found to evade the immune system by employing numerous mechanisms and one such mechanism is the activation of negative regulators, PD-1 and CTLA-4 [13]. High expressions of ligands that are specific to the negative regulatory receptors have been detected on the cancer and immune cells in the tumor microenvironment [14, 15]. Further IFN-γ produced by the T cell induces the expression of these inhibitory ligands on the cells of the tumor microenvironment [16–18]. Consequently, T cells receiving high level of inhibitory signals become inactive and have suppressed effector functions. PD-1 and CTLA-4 are extensively being studied and are considered as potential targets for activating the tumor infiltrating T cells that remain inactive in the immunosuppressive tumor microenvironment [19, 20]. Antibodies against these receptors have shown exceptional efficacy and are considered as promising drugs that could potentially revolutionize cancer treatment. A few of the antibodies for instance, Nivolumab and Pembrolizumab targeting PD-1 receptor have been approved by the FDA (Food and Drug administration) for the treatment of melanoma [2]. However, administration of these immune checkpoint inhibitor drugs has numerous adverse effects and the treatment remains ineffective for a significant proportion of patients [21]. Apart from its role in inducing tumor immune escape, its role in several viral infections such as HIV (Human immunodeficiency virus), HCV (Hepatitis C virus) and HBV (Hepatitis B virus) are also demonstrated [22]. Exhaustion of T cells due to persistent TCR stimulation is observed during chronic viral infections [23, 24]. Hence, an understanding of how the PD-1 receptor influences the T cell response is crucial for the development of effective treatment against cancer, autoimmunity and several other diseases.

Mathematical models have been an integral part in understanding complex biological phenomena such as apoptosis [25], cell cycle [26, 27], NF-κB oscillations [28], cellular differentiation [29], cell signaling [30]. Mathematical modelling tools have become popular in explaining various aspects of immune systems[31] such as discrimination of self and non-self antigen [32, 33], T cell activation [34–36], cytokine signaling pathways [37–39], T cell differentiation[40]. With the accumulation of quantitative and semi quantitative experimental results, modeling the TCR signaling network is increasingly being explored [41]. Protein-protein docking, molecular dynamics and mathematical modeling studies on interaction of PD-1 with its ligands have provided insights into the atomistic details and factors affecting these interactions at cellular interfaces [42, 43]. Mathematical models were developed to predict tumor response to immune checkpoint inhibitors, and immune checkpoint therapy in combination with radiotherapy [44, 45]. However, until now there is no mathematical model available to understand the influence of PD-1 on TCR signaling molecules.

To better understand the molecular basis of PD-1 induced inactivation of T cell signaling molecules, we constructed a deterministic mathematical model of PD-1 regulatory pathway. The proposed model investigates the role of feedback regulatory mechanisms in inhibition of early TCR signaling by PD-1. The model simulations provides the mechanistic basis of several features of PD-1 mediated inhibition that were observed in the recent experiments done using the reconstitution system by Hui et al [46].

## Results and discussion

### Review of TCR and PD-1 activation pathway

TCR is stimulated by the binding of cognate p-MHC (peptide-major histocompatibility complex) present on the surface of antigen-presenting cells. Stimulation of TCR results in the phosphorylation of cytoplasmic domain of the TCR complex at the ITAMs (Immunoreceptor tyrosine-based activation motifs) by the Src family tyrosine kinase, Lck (Lymphocyte specific protein tyrosine kinase). Phosphorylated CD3 ITAMs recruit Zap70 (Zeta-chain-associated protein of 70 KDa), a Syk family kinase. Studies on ZAP70 phosphorylation have shown that Y315 and Y319 are initially phosphorylated by Lck and this in turn allows the Y493 present in the activation loop of the Zap70 to get phosphorylated. Phosphorylation of Y493 has shown to be crucial for the complete activation of the kinase Zap70 [47, 48]. Activated Zap70 phosphorylates TCR signaling molecules such as the adaptor molecule, LAT (Linker for activation of T cells) and Slp76 (SH2-domain-containing leukocyte protein of 76 KDa)[49]. Phosphorylated LAT recruits signaling proteins such as Gads (Grb2-related adapter protein 2), Grb2 which in turn interact with other signaling proteins. Gads associates with Slp76 and this brings Slp76 in close proximity to activated Zap70 bound to the cytoplasmic domain of TCR complex. Several other molecules are recruited to form a complex called as signalosome and the signal is transmitted to the downstream effector molecules. Signal is transduced to the nucleus by the activation of the transcription factors AP1, NFAT and NF-KB resulting in changes of the gene expression [50–53]. CD28 receptor is activated upon binding to its ligands B7-1 and B7-2 inducing a conformational change in its cytosolic domain. This recruits PI3K and it transmits signal by activating other signaling molecules[54].

PD-1 receptor is stimulated by binding to its ligand, PD-L1 or PD-L2 expressed on the surface of APCs (Antigen Presenting Cells) [55]. Studies have demonstrated the inhibitory effect of PD-1 stimulation on several of the TCR signaling components [56, 57]. PD-1 receptor upon activation recruits the cytosolic tyrosine phosphatase Shp2, which in turn dephosphorylates the TCR and CD28 signaling components. Recently, a FRET (Fluorescence Resonance Energy Transfer) based assay was done by Hui et al. [46] using a biochemical reconstitution system, to understand the molecular basis of suppression of T cell functions by PD-1. Few of the components of the T cell signaling network were reconstituted to understand the effect of PD-1 activation on the sequence of events that occur after the phosphorylation of cytosolic domain of the receptors TCR and CD28. In the *in vitro* reconstitution system, the cytosolic domains of receptors and membrane bound proteins were tethered to the surface of LUVs (large unilamellar vesicles) and the cytosolic signaling components were added to the solution. FRET based techniques were used to quantify the phosphorylation state of the receptors involved. These experiments demonstrated that Shp2 recruited by the cytosolic domain of PD-1 receptor can directly dephosphorylate the cytosolic tails of CD3ζ and CD28. The key findings of the experiments are that the CD28 is preferentially dephosphorylated in response to increasing PD-1 concentration whereas the CD3ζ chain and the other components of the TCR signaling tested are less sensitive to the inhibitory effect of PD-1. Further these findings were also confirmed using a cell based experiment employing Jurkat T cells and Raji B cells.

### Model construction

**Fig 1:**
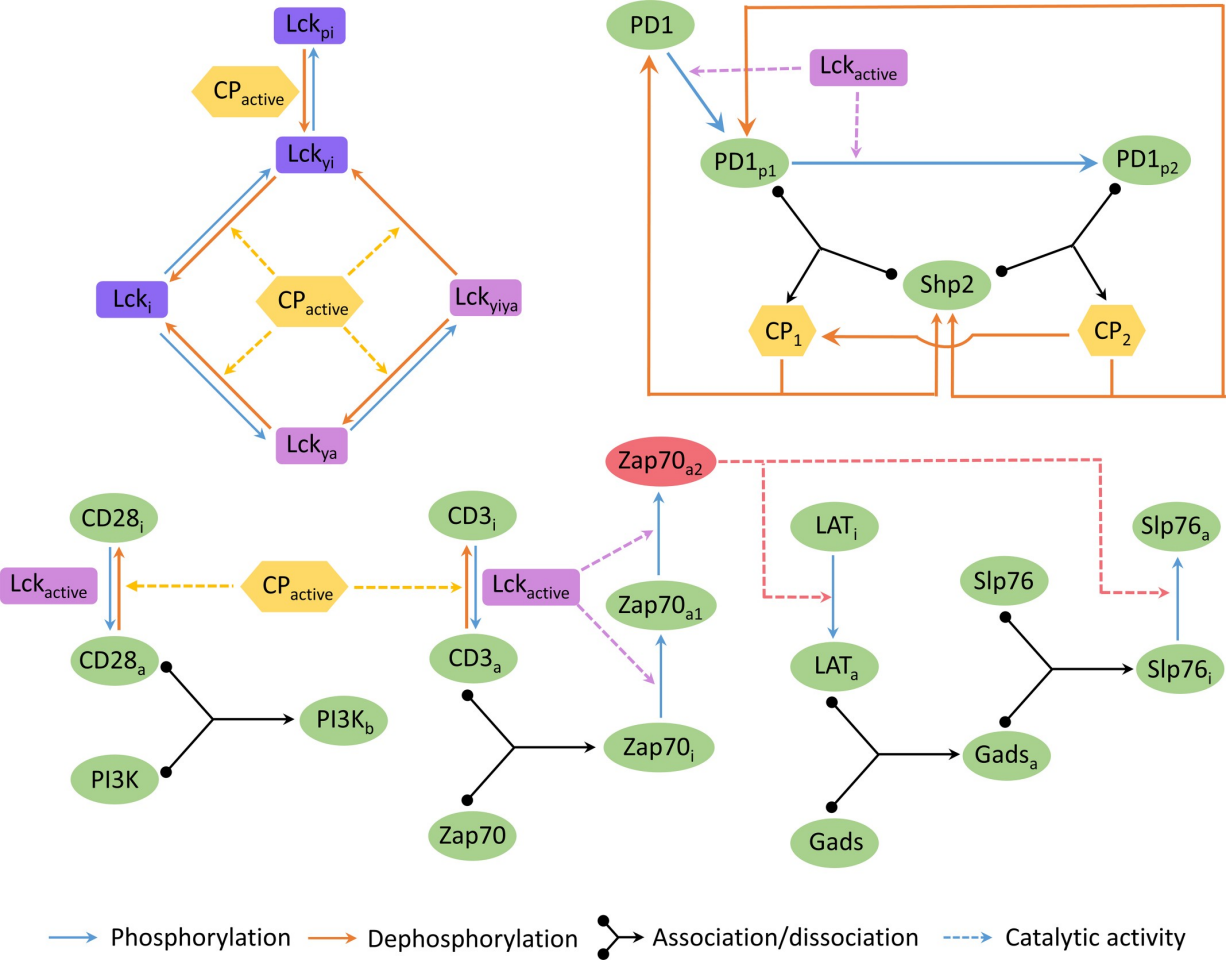
Network diagram of the model of PD-1 signaling pathway. Solid line represents a chemical reaction and dashed line represents catalytic effect on a reaction. Three types of chemical reactions are involved in the model: phosphorylation, dephosphorylation and association-dissociation. Lck_active_ (= Lck_ya_+Lck_yiya_) is the total active form of Lck and similarly CP_active_ (= CP_1_+CP_2_) is the total Shp2 bound to PD-1. The model equations and parameters are given in Table 1 and Table 2. The description of model variables are listed in Table A in S1 File.

We have constructed an ordinary differential equation (ODE) based mathematical model for the early signaling events following the TCR, CD28 and PD-1 stimulation. The full network diagram of the model is given in Fig 1 and a simplified influence diagram of the model can be found in Figure A in S1 File.

**Table 1:** List of model equations.

$\frac{dCD3_{a}}{dt}=\frac{k_{p,cd3}∙Lck_{active}∙\left(CD3_{T}-\left(CD3_{a}+Zap70_{i}+Zap70_{a1}+Zap70_{a2}\right)\right)}{K_{Mp,cd3}+\left(CD3_{T}-\left(CD3_{a}+Zap70_{i}+Zap70_{a1}+Zap70_{a2}\right)\right)}-\frac{k_{dp,cd3}∙CP_{active}∙CD3_{a}}{K_{Mdp,cd3}+CD3_{a}}+k_{d,zap}∙Zap70_{i}-k_{a,zap}∙CD3_{a}∙\left(Zap70_{T}-\left(Zap70_{i}+Zap70_{a1}+Zap70_{a2}\right)\right)$
$\frac{dCD28_{a}}{dt}=\frac{k_{p,cd28}∙Lck_{active}∙(CD28_{T}-(CD28_{a}+PI3K_{b}))}{K_{Mp,cd28}+(CD28_{T}-(CD28_{a}+PI3K_{b}))}-\frac{k_{dp,cd28}∙CP_{active}∙CD28_{a}}{K_{Mdp,cd28}+CD28_{a}}+k_{d,pi3k}∙PI3K_{b}-k_{a,pi3k}∙CD28_{a}∙(PI3K_{T}-PI3K_{b})$
$\frac{dPD1_{p1}}{dt}=\frac{k_{p,pd1}∙Lck_{active}∙PD1}{K_{Mp,pd1}+PD1}*(1-\frac{PD1_{p1}+PD1_{p2}}{Lck_{T}k})-\frac{k_{p,pd1}∙PD1_{p1}∙Lck_{active}}{K_{Mp,pd1}+PD1_{p1}}-k_{a,shp}∙PD1_{p1}∙Shp2+k_{d1,shp}∙CP_{1}+k_{d2,shp}∙CP_{2}$
$\frac{dPD1}{dt}=-\frac{k_{p,pd1}∙Lck_{active}∙PD1}{K_{Mp,pd1}+PD1}(1-\frac{PD1_{p1}+PD1_{p2}}{Lck_{T}k})+k_{d2,shp}∙CP_{1}$
$\frac{dPD1_{p2}}{dt}=\frac{k_{p,pd1}∙PD1_{p1}∙Lck_{active}}{K_{Mp,pd1}+PD1_{p1}}-k_{a,shp}∙PD1_{p2}∙Shp2+k_{d1,shp}∙CP_{2}$
$\frac{dShp2}{dt}=-k_{a,shp}∙Shp2∙(PD1_{p1}+PD1_{p2})+k_{d1,shp}∙(CP_{1}+CP_{2})+k_{d2,shp}∙(CP_{1}+CP_{2})$
$\frac{dCP_{1}}{dt}=k_{dp,cp2}∙CP_{2}+k_{a,shp}∙PD1_{p1}∙Shp2-k_{d1,shp}∙CP_{1}-k_{d2,shp}∙CP_{1}$
$\frac{dCP_{2}}{dt}=-k_{dp,cp2}∙CP_{2}+k_{a,shp}∙PD1_{p2}∙Shp2-k_{d1,shp}∙CP_{2}-k_{d2,shp}∙CP_{2}$
$\frac{dLck_{yiya}}{dt}=-k_{dpa,yiya}∙CP_{active}∙Lck_{yiya}-k_{dpi,yiya}∙CP_{active}∙Lck_{yiya}+k_{pi,ya}∙Lck_{ya}$
$\frac{dLck_{yi}}{dt}=k_{dpa,yiya}∙CP_{active}∙Lck_{yiya}-k_{dpi,yi}∙CP_{active}∙Lck_{yi}+k_{pi,i}∙\left(Lck_{T}-(Lck_{yiya}+Lck_{yi}+Lck_{ya}+Lck_{pi})\right)-k_{pa,yi}∙Lck_{yi}+k_{dpa,pi}∙CP_{active}∙Lck_{pi}$
$\frac{dLck_{ya}}{dt}=k_{dpi,yiya}∙CP_{active}∙Lck_{yiya}-k_{pi,ya}∙Lck_{ya}-k_{dpa,ya}∙CP_{active}∙Lck_{ya}+k_{pa,i}∙\left(Lck_{T}-(Lck_{yiya}+Lck_{yi}+Lck_{ya}+Lck_{pi})\right)$
$\frac{dLck_{pi}}{dt}=-k_{dpa,pi}∙CP_{active}∙Lck_{pi}+k_{pa,yi}∙Lck_{yi}$
$\frac{dZap70_{i}}{dt}=k_{a,zap}∙CD3_{a}∙(Zap70_{T}-(Zap70_{i}+Zap70_{a1}+Zap70_{a2}))-k_{d,zap}∙Zap70_{i}-k_{p1,zap}∙Lck_{active}∙Zap70_{i}$
$\frac{dZap70_{a1}}{dt}=k_{p1,zap}∙Lck_{active}∙Zap70_{i}-k_{p2,zap}∙Lck_{active}∙Zap70_{a1}$
$\frac{dZap70_{a2}}{dt}=k_{p2,zap}∙Lck_{active}∙Zap70_{a1}$
$\frac{dLAT_{a}}{dt}=k_{p,lat}∙Zap70_{a2}∙(LAT_{T}-(LAT_{a}+Gads_{a}+Slp76_{i}+Slp76_{a}))-k_{a,gads}∙LAT_{a}∙(Gads_{T}-(Gads_{a}+Slp76_{a}+Slp76_{i}))+k_{d,gads}∙Gads_{a}$
$\frac{dSlp76_{i}}{dt}=k_{a,slp}∙Gads_{a}∙(Slp76_{T}-(Slp76_{i}+Slp76_{a}))-k_{d,slp}∙Slp76_{i}-k_{p,slp}∙Slp76_{i}∙Zap70_{a2}$
$\frac{dSlp76_{a}}{dt}=k_{p,slp}∙Slp76_{i}∙Zap70_{a2}$
$\frac{dGads_{a}}{dt}=k_{a,gads}∙LAT_{a}∙\left(Gads_{T}-(Gads_{a}+Slp76_{i}+Slp76_{a})\right)-k_{d,gads}∙Gads_{a}-k_{a,slp}∙Gads_{a}∙\left({Slp76}_{T}-(Slp76_{i}+Slp76_{a})\right)+k_{d,slp}∙Slp76_{i}$
$\frac{dPI3K_{b}}{dt}=k_{a,pi3k}∙CD28_{a}∙(PI3K_{T}-PI3K_{b})-k_{d,pi3k}∙PI3K_{b}$
*Lck*_*active*_ = *Lck*_*yiya*_ + *Lck*_*ya*_ *CP*_*active*_ = *CP*_1_ + *CP*_2_

**Table 2:** Description of model parameters and their values.

Kinetic rate constants				
	Parameter	Description of rate constant	Value used in the model (nM.s)^-1^	Literature value
1	*k*_*dpa*,*yiya*_	Dephosphorylation of Y394 from Lck_yiya_ by CP_1_ and CP_2_	2.4×10^−5^	-
2	*k*_*dpi*,*yi*_	Dephosphorylation of Y505 from Lck_yi_ by CP_1_ and CP_2_	2.4×10^−5^	-
3	*k*_*dpi*,*yiya*_	Dephosphorylation of Y505 from Lck_yiya_ by CP_1_ and CP_2_	1.2×10^−5^	-
4	*k*_*dpa*,*ya*_	Dephosphorylation of Y394 from Lck_ya_ by CP_1_ and CP_2_	6×10^−6^	-
5	*k*_*dpa*,*pi*_	Dephosphorylation of Y394 from Lck_pi_ by CP_1_ and CP_2_	1.2×10^−7^	-
6	*k*_*a*,*zap*_	Association of CD3_a_ and Zap70	7×10^−5^	3.5×10^−5^–9.1×10^−5^ (nM.s)^-1^[58]
7	*k*_*p*1,*zap*_	Phosphorylation of Zap70 Y315 in Zap70_i_ by Lck	2×10^−6^	-
8	*k*_*p*2,*zap*_	Phosphorylation of Zap70 Y493 in Zap70_a1_by Lck	3×10^−5^	-
9	*k*_*p*,*lat*_	Phosphorylation of LAT_i_ by Zap70_a2_	10^−3^	-
10	*k*_*a*,*slp*_	Association of SLP76 and Gads_a_	1.5×10^−2^	1.5×10^−2^ (nM.s)^-1^ [59]
11	*k*_*p*,*slp*_	Phosphorylation of SLP76_i_ by Zap70_a2_ to form SLP76_a_	0.003	-
12	*k*_*a*,*gads*_	Association of LAT_a_ and Gads to form Gads_a_	5×10^−4^	-
13	*k*_*a*,*pi*3*k*_	Association of CD28_a_ and PI3K	1.4×10^−6^	-
14	*k*_*a*,*shp*_	Association of phosphorylated PD1 and Shp2	6.5×10^−3^	10^−3^(nM.s)^-1^ [60, 61]
	**Parameter**	**Description of rate constant**	**Value used in the model (s**^**-1**^**)**	**Literature value**
15	*k*_*pi*,*i*_	Auto-phosphorylation of Y505 of Lck_i_	6×10^−7^	[62]
16	*k*_*pi*,*ya*_	Auto-phosphorylation of Y505 of Lck_ya_	6×10^−5^	[62]
17	*k*_*pa*,*i*_	Auto-phosphorylation of Y394 of Lck_i_	1×10^−6^	[62]
18	*k*_*pa*,*yi*_	Auto-phosphorylation of Y394 of Lck_yi_	7.5×10^−4^	[62]
19	*k*_*p*,*cd*3_	Phosphorylation of CD3_i_ by Lck	3.29	1–7 s^-1^ [63]
20	*k*_*dp*,*cd*3_	Dephosphorylation of CD3_a_ by CP_1_ and CP_2_	5	-
21	*k*_*d*,*zap*_	Dissociation of Zap70_i_	10^−3^	1.4×10^−4^–9×10^−4^ s^-1^ [58]
22	*k*_*d*,*slp*_	Dissociation of SLP76_i_ to Gads_a_ and SLP76	0.12	0.12 s^-1^[59]
23	*k*_*d*,*gads*_	Dissociation of Gads_a_ into LAT_a_ and Gads	1.5	-
24	*k*_*p*,*cd2*8_	Phosphorylation of CD28_i_ by Lck	1	-
25	*k*_*dp*,*cd*28_	Dephosphorylation of CD28_a_ by CP_1_ and CP_2_	5	-
26	*k*_*d*,*pi*3*k*_	Dissociation of PI3K_b_ into CD28_a_ and PI3K	9×10^−4^	-
27	*k*_*p*,*pd*1_	Phosphorylation of PD1 by Lck	7.5	-
28	*k*_*d*1,*shp*_	Dissociation of CP_1_ into PD1_p1_ and Shp2 or CP_2_ into PD1_p2_ and Shp2	10	10 s^-1^ [61, 64, 65]
29	*k*_*dp*,*cp*2_	Self dephosphorylation of CP_2_ to form CP_1_	5×10^−8^	-
30	*k*_*d*2,*shp*_	Dissociation of CP_2_ into PD1_p1_ and Shp2 or CP_1_ into PD1 and Shp2 due to self dephosphorylation	1	1 s^-1^ [61, 64, 65]
**Michaelis-Menten constants**				
	**Parameter**	**Description of rate constant**	**Value used in the model (nM)**	**Literature value**
31	*K*_*Mp*,*cd*3_	Phosphorylation of CD3_i_ by Lck	80	69–172 nM[63]
32	*K*_*Mdp*,*cd*3_	Dephosphorylation of CD3_a_ by CP_1_ and CP_2_	150	-
33	*K*_*Mp*,*cd*28_	Phosphorylation of CD28_i_ by Lck	1000	-
34	*K*_*Mdp*,*cd*28_	Dephosphorylation of CD28_a_by CP_1_ and CP_2_	500	-
35	*K*_*Mp*,*pd*1_	Phosphorylation of PD1 and PD1_p1_ by Lck to form PD1_p1_and PD1_p2_ respectively.	1000	-
**Constant used in the correction factor**				
36	*k*	-	41	-

Ligated PD-1 receptor is doubly phosphorylated in distributive manner by the active Lck kinase (Lck_active_). Shp2 binds to both the singly and doubly phosphorylated PD-1 to form the complexes referred as CP_1_ and CP_2_ respectively. Shp2 bound to the phosphorylated PD-1 (CP_2_and CP_1_) distributively self-dephosphorylate the PD-1 to return either CP_1_ or mono/unphosphorylated PD-1 and thereby releasing Shp2 from the complex. CD28 and CD3ζ receptors are phosphorylated by the active Lck kinase and dephosphorylated by the Shp2 bound to the phosphorylated PD-1 (CP_1_ and CP_2_). We must mention here that free Shp2 does not have any catalytic activity. We used Michaelis-Menten kinetics to model these phosphorylation and dephosphorylation reactions.

Phosphorylation of CD28 and CD3ζ lead to engagement of free PI3K and free Zap70 to form PI3K_b_ andZap70_i_complexes, respectively. Zap70 bound to CD3ζ (Zap70_i_) is phosphorylated by active Lck at tyrosine Y315 initially to form Zap70_a1_ and then at tyrosine Y493 to form Zap70_a2_. Zap70_a2_ is considered as the active form of Zap70 and it phosphorylates LAT, referred as LAT_i_, to form phosphorylated LAT, referred as LAT_a_. Although LAT_i_ is phosphorylated at several sites by active Zap70, the individual phosphorylation events of LAT molecule are not considered separately in the model to reduce the complexity and therefore these phosphorylations are considered as processive in nature. LAT_a_ is considered to be the active form of LAT and it binds to the adaptor protein Gads to form the complex Gads_a_. Gads_a_ in turn interacts with free Slp76 to form LAT_a_-Gads-Slp76 complex referred as Slp76_i_. Slp76 bound to the LAT_a_-Gads complex is phosphorylated by Zap70_a2_ to form the complex Slp76_a_.

Experimental and molecular dynamics studies have shown that phosphorylation of Lck at Y394 has an activating effect and whereas Y505 is inhibitory[66]. Further it is believed that when the Lck is phosphorylated at Y505 first, it acquires a closed conformation and subsequent phosphorylation at Y394 does not change its activity [67]. Therefore five different forms of Lck are considered in the model depending on the phosphorylation state of the two tyrosine amino acid residues (Y394 and Y505): a. unphosphorylated at both tyrosines (Lck_i_), b. phosphorylated only at Y394 (Lck_ya_), c. phosphorylated only at Y505 (Lck_yi_), d.Y394 and Y505 phosphorylated sequentially (Lck_yiya_) and e. Y505 and Y394 phosphorylated consecutively (Lck_pi_). Consequently, among all forms of Lck in the model, only Lck_ya_ and Lck_yiya_ were considered to be active and are capable of phosphorylating their substrates. All these reactions are modeled using mass action rate law of chemical reactions.

Experimental results by Hui et al[46] on the recruitment of Shp2 at various doses of PD-1 demonstrated that Shp2 recruitment to the PD-1 saturates beyond a certain concentration of PD-1. As the mechanistic basis to this observation is not known, a correction factor was introduced in the equations for the phosphorylation of PD-1 to account for this observation. This correction factor ensures the saturation of Shp2 recruitment at increasing concentration of PD-1, depending on the concentration of the Lck in the system.

### Model parameterization and simulation

The model consists of 20 ODEs and 36 parameters. The model equations, the parameters values and the biological description of variables of the model are listed in the Table 1, Table 2 and Table A in S1 File respectively. Among 36 parameters we managed to get values for 13 parameters from experimental literature. Rest of the rate constants were parameterized to reproduce the experimental data of Hui et al [46]. Shp2 may bind to doubly phosphorylated PD-1 with higher affinity as compared to the singly phosphorylated PD-1. However due to lack of experimental binding data and also to reduce number of parameters we preferred same binding parameters for both the phosphorylated forms. CP_1_ and CP_2_ are assumed to have same catalytic activity in dephosphorylation of all of its substrates. For doubly phosphorylated Lck, dephosphorylation of Y394 is faster than that of Y505. Such preferential dephosphorylation of activating tyrosine by SHP-1 has been observed earlier[68]. CP_1_ and CP_2_ are also considered to be equally stable. However, rate constants for the auto-phosphorylation of different forms of Lck were assumed to be different. Lck auto-phosphorylation rate constants have been estimated and employed in a published mathematical model by Rohrs et al [62]. The catalytic rate estimated by Rohrs et al for the tyrosines Y394 and Y505 differ several orders of magnitude depending on the Lck form that trans-autophosphorylates Lck. Although, the autophosphorylations of a given Lck form by different forms are not distinguished in the model, the rate constants employed in our model are well within the range of parameters used by Rohrs et al.

The system of equations is solved with MATLAB using ode15s solver. Initial values for the model components are based on the concentration of components used in the experiments by Hui et al [46]. Simulations for a few experiments for which the concentrations are not mentioned, are done by using guessed concentrations. However, changing the concentration in most cases did not alter the qualitative behavior of simulated responses. Initial concentration for the following Lck states–Lck_yiya_, Lck_ya_, Lck_yi_ and Lck_i_ are taken as 25% of the total Lck concentration. This is consistent with the relative proportion of different forms of Lck measured in Jurkat T cells [67]. The initial condition of Lck_pi_ was chosen to be 0 as it is believed that majority of doubly phosphorylated Lck is derived by the phosphorylation of Lck_ya_ at Y505 [67].

Units of concentration and time in the model are nM and seconds, respectively. Hence, initial concentrations of species were provided in nM. In few cases, nM concentrations in the simulation results were converted to molecules/μm^2^ for easier quantitative comparison with published experimental results. Michaelis-Menten constant of phosphorylation of CD3ζ by Lck obtained from the literature had unit of μm^-2^ and was converted to nM before employing in the model. To interconvert the concentration units between nM and molecules/μm^2^we used 1 nM = 2.9 molecules/μm^2^as conversion factor. Concentration as surface density (molecules/μm^2^) is estimated by dividing the number of molecules of a species (calculated from concentration in nM) by the total area of exposed vesicle membrane [63]. The surface density of protein (*d*) is related to the number of protein molecules (*N*) and surface area of liposome (*σ*) as *d* = *N*/*σ*. The *N* and *σ* are given by, *N* = [*P*]*VN*_*A*_ and *σ* = [*L*]*VN*_*A*_*fa*_*L*_, where [*P*], [*L*], *V*, *N*_*A*_, *f* and *a*_*L*_ are concentration of protein, concentration of phospholipid, volume, Avogadro’s number, fraction of exposed lipid and the area of each lipid head. For a liposome of diameter 200 nm, ~52.6% of total lipids were found to present on the outer membrane of liposome [63]. Thus with a lipid concentration of 1 mM (as used by Hui *et al*. [46]), the protein concentration of 1 nM becomes equivalent to surface density of ~2.9 molecules/μm^2^.

### Model validation

We benchmarked our model by performing several simulations corresponding to the experiments done by Hui et al on the biochemical reconstitution system. It includes the time course of recruitment of Shp2, Zap70 and PI3k to PD-1, CD3ζ and CD28 respectively; comparison of phosphorylation activity of Lck on CD3ζ and CD28; dissociation of Zap70 and PI3k from their corresponding receptors in the absence of Lck activity; the dephosphorylation of TCR signaling molecules and CD28 due to PD-1.

**Fig 2:**
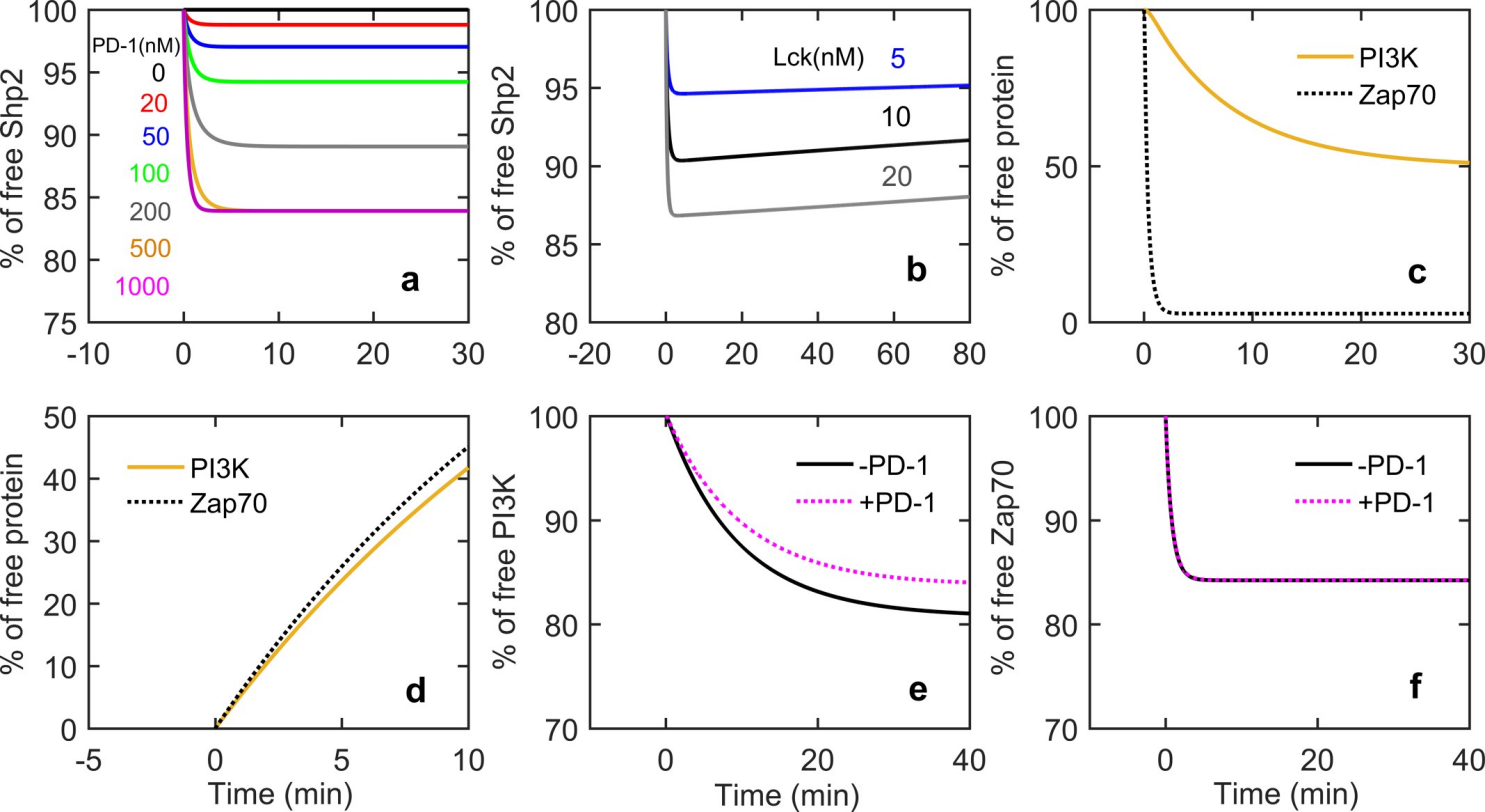
a) Time course of Shp2 (binding domain) recruitment for different concentrations of PD-1 receptor with 7.2 nM Lck and 100 nM Shp2. b) Time course of full length Shp2 recruitment by PD-1 receptor for different Lck concentrations with 300nM PD-1 and 50 nM Shp2. c) Time course of PI3K and Zap70 recruitment in absence of PD1 with 800 nM CD3ζ and CD28, 300nM Zap70 and PI3k, and 100nM Lck. d) Time course of PI3K and Zap70 disengagement in absence of Lck and PD1 with 300nMof Zap70_i_, 300nMof PI3K_b_, and 200nMof CP_2_. e) and f) Effect of PD-1 on the time course of recruitment of PI3K and Zap70 respectively. For e and f the concentrations used were 50 nM CD3ζ, 300 nM Zap70, 250 nM CD28, 500 nM PI3K, 300 nM Lck, 100 nM PD-1 and Shp2.

We investigated the recruitment of Shp2 to the PD-1 receptor upon its phosphorylation by Lck. Upon introduction of Lck at time zero, Shp2 rapidly binds to PD-1 and the extent of binding increases with PD-1 concentration (Fig 2A). Here we did not allow Shp2 to dephosphorylate the receptor after binding in order to recapture the experiments consisting of only binding domain of Shp-2 lacking catalytic activity. In order to determine the effect of self-dephosphorylation of PD-1 receptor bound to Shp2 on the kinetics of Shp2 recruitment, we allowed self-dephosphorylations of CP_1_ and CP_2_ complexes. Results from Fig 2B indicate that after a rapid initial engagement, Shp2 slowly dissociates from the PD-1 due to the dephosphorylations. Further there is a weak dependence of Lck on the dissociation kinetics of Shp2 as the slope of the line increases slightly with the increased Lck concentrations.

Next we investigated the kinetics of Lck phosphorylation and PD-1 mediated dephosphorylation of receptors in absence of each other. We determined the kinetics of engagement of PI3K and Zap70 to their respective receptors upon Lck mediated phosphorylation of CD28 and CD3ζ in absence of PD-1 (Fig 2C). Similar to the experimental profile, simulation results show that phosphorylation of CD3ζ and subsequent recruitment of Zap70 proceeds at a much higher rate as compared to the CD28 phosphorylation and subsequent PI3K recruitment. Further we investigated the PD-1 receptor mediated dephosphorylation dynamics of CD28 and CD3ζ by calculating the percentage of free PI3K and Zap70 in absence of Lck (Fig 2D). Here we started simulations with a certain initial concentrations of Zap70_i_ and PI3K_b_ considering that all of the CD28 and CD3ζ are already phosphorylated leading to full engagement of PI3K and Zap70 respectively. The initial concentrations of Lck and PD-1 were also set to zero. Then to determine the dephosphorylation kinetics we used a non-zero concentration of CP_2_ complex as a proxy for membrane bound Shp2 used in the experimental protocol. In order to be consistent with the experimental protocol we did not allow self-dephosphorylation, dissociation and the degradation of CP_2_ complex. In experiments Hui et al used full length Shp2 protein and it was directly attached to the membrane of vesicles. As observed in the experiments, simulations results showed that the percentage of free PI3K and Zap70 increases monotonically with time with similar rates.

Till now we showed the kinetics of phosphorylations or dephosphorylations in absence of either PD-1 or Lck. Now we investigated the kinetics when both of them are present essentially simulating the whole network. Simulation results showed that in presence of PD-1 the steady state values of free PI3K and Zap70 are higher than in absence of PD-1 (Figure B in S1 File). This is due to dephosphorylations of CD28 and CD3ζ resulting release of respecting signaling molecules. The kinetics of PI3K indicates that the effect of PD-1 on CD28 is drastic as compared to CD3ζ (Fig 2E and 2F, Figure B in S1 File). We must point that all the simulation results of Fig 2 are in excellent qualitative and quantitative agreement with the experimental plots reported in Hui et al [46].

**Fig 3:**
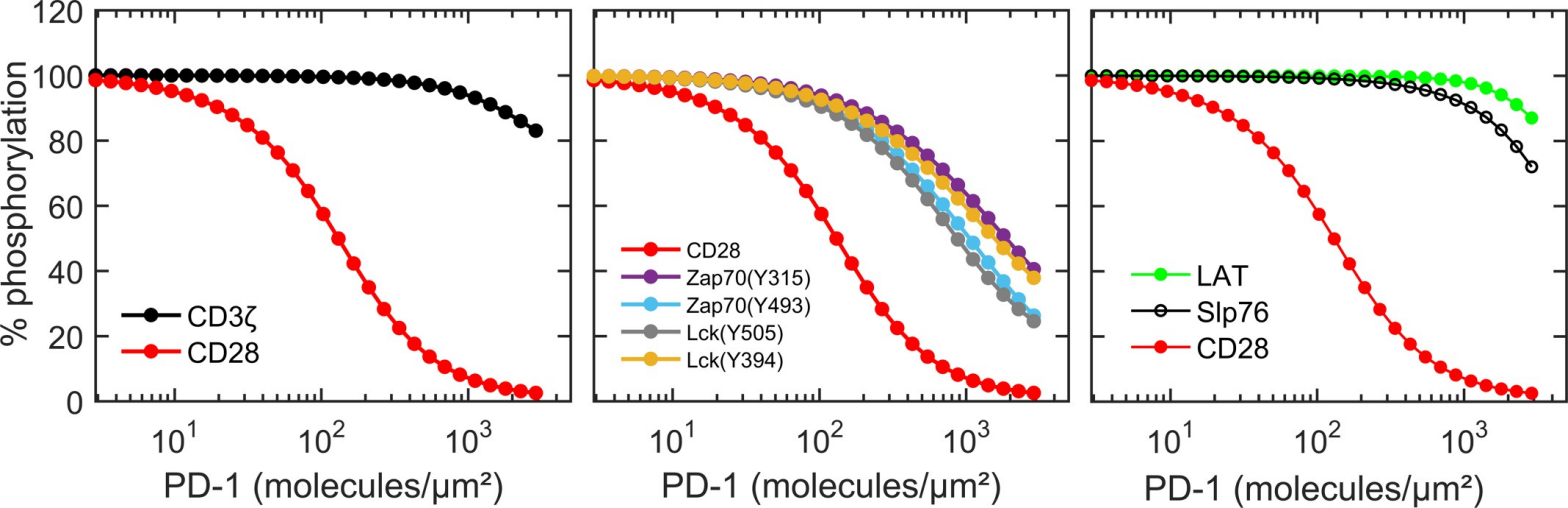
PD-1 dose response curves of various signaling molecules. Concentrations of the components are as follows: 100nM Lck and CD3ζ; 200nM PI3K; 300nM CD28, Zap70, Shp2, LAT, Gads and SLP76. These concentrations are same as Hui et al [46]. Concentration of phosphorylated species is calculated as: Phosphorylated CD3ζ = CD3_a_+Zap70_i_+Zap70_a1_+Zap70_a2_, Phosphorylated CD28 = CD28_a_+PI3K_b_, ZAP70 phosphorylated at Y315 = Zap70_a1_+Zap70_a2_, Zap70 phosphorylated at Y493 = Zap70_a2_, Lck phosphorylated at Y505 = Lck_yi_+Lck_yiya_+Lck_pi_, Lck phosphorylated at Y394 = Lck_ya_+Lck_yiya_+Lck_pi_, Phosphorylated LAT = LAT_a_+ Gads_a_+Slp76_i_+Slp76_a_ and Phosphorylated SLP76 = Slp76_a_.

We now turn our focus towards the steady state results of model simulations and their comparison with reported experimental literature. We have carried out PD-1 dose response simulations of the full model where at a given concentration of PD-1 we record response at 30 min (Fig 3) as done in the experiments. Concentration of phosphorylated forms of each species was calculated and normalized with respect to the phosphorylation in the absence of PD-1. Phosphorylated CD3ζ was calculated by adding the CD3_a_, Zap70_i_, Zap70_a1_ and Zap70_a2_ in the model. Similarly, phosphorylated CD28 includes CD28_a_ and PI3K_b_; phosphorylated LAT includes LAT_a_, Gads_a_, Slp76_i_ and Slp76_a_. Lck phosphorylation at Y394 is calculated by adding Lck_yiya_, Lck_ya_, and Lck_pi_. Similarly, Lck phosphorylated at Y505 was calculated by adding Lck_yiya_, Lck_yi_ and Lck_pi_.

Consistent with the experimental results PD-1 leads to dramatic decrease in CD28 phosphorylation. Whereas in the same concentration range of PD-1, dephosphorylation of CD3ζ is not significant (Fig 3). We have calculated the IC_50_ and Hill coefficient of the response by fitting the dose response curves with a standard Hill function ($k∙x^{n}/\left(IC_{50}^{n}+x^{n}\right)$ where *k* is the maximum response). See Figure C in S1 File for fitting of dose responses with Hill function. The IC_50_ value of CD28 is smaller in several orders of magnitude than that of CD3ζ highlighting the sensitivity of CD28 towards PD-1 (Table 3). The dose response curves for individual phosphorylations of Lck show similar IC_50_ values indicating the identical effects of PD-1 on dephosphorylating tyrosines Y394 and Y505 of Lck. Further Y315 and Y493 tyrosines of Zap70 have almost similar IC_50_ values in PD-1 dose responses. Two other key downstream signaling molecules in CD3ζ pathway, LAT and Slp76, showed less susceptibility towards PD-1 dephosphorylation. Overall dose response results indicate CD28 is more potent target of PD-1 as compared to CD3ζ and Zap70 is stronger target among the target molecules in CD3ζ pathway.

**Table 3:** Comparison of model calculated and experimental [46] IC_50_ values for PD-1 dose response curves.

Signaling molecule	Hill coefficient	IC_50_ (PD-1 molecules/μm^2^)	Experimental IC_50_ (PD-1 molecules/μm^2^)
CD3ζ	1.40	3017.4	>3000
CD28	1.25	126.3	96
Lck (Y505)	1.15	680.0	400
Lck (Y394)	1.05	980.7	~600
Zap70 (Y315)	1.04	1285.0	~3000
Zap70 (Y493)	1.07	919.1	~1400
LAT	2.09	4698.6	>3000
SLP76	1.62	3779.0	~3000

### Insights and predictions

**Fig 4:**
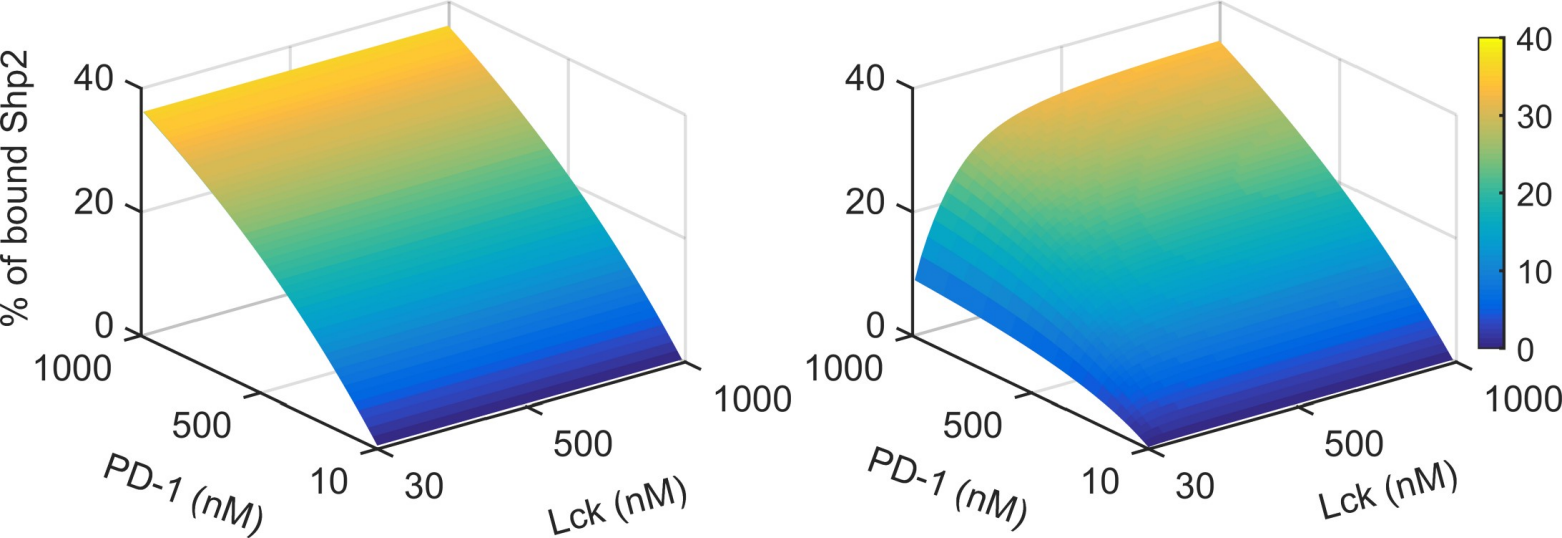
**Recruitment of Shp2 after 30 min of simulation for various doses of Lck and PD-1 with 300 nM of Shp2 without (left) and with (right) self-dephosphorylation of PD-1 by Shp2**.

In Figs 2 and 3 we have shown results from the model that quantitatively recaptures the experimental observations reported in Hui et al and thus validating the model. Hui et al suggested that the net dissociation of Shp2 from PD-1 complexes seen in the experiments, is due to the dephosphorylation of PD-1 by the PD-1-Shp2 complex. To test this, we performed simulations in the presence and absence of self dephosphorylation activity of CP_1_ and CP_2_ complexes for a large range of PD-1 and Lck concentrations. We find that till 500 nM of Lck there is a net decrease in Shp2 binding with increase in PD-1 concentration (Fig 4) as compared to simulations lacking self-dephosphorylation of PD-1 receptor by Shp2.

We generated similar surface plots to determine the dependence of phosphorylation of CD28 and CD3ζ and the subsequent recruitment of PI3K and Zap70 by the respective phosphorylated receptors in absence of PD-1 (Figure D in S1 File). We find CD28 and CD3ζ are completely phosphorylated irrespective of the substrate concentration for Lck concentrations above 10 nM due to the large catalytic activity of Lck. This also suggests that Lck can completely phosphorylate all the CD28 and CD3ζ at physiological concentration of these components. Consequently PI3K recruitment and Zap70 recruitment are almost independent of Lck concentration. In the case of Zap70, 100% recruitment is achieved at approximately 500nM of CD3ζ concentration. Percentage of active Zap70 increases with increasing CD3ζ and Lck concentrations thus showing stronger dependence on CD3ζ and Lck. As opposed to this, complete recruitment and activation of Slp76 is achieved at low Lck and CD3ζ concentrations (Figure D in S1 File).

**Fig 5:**
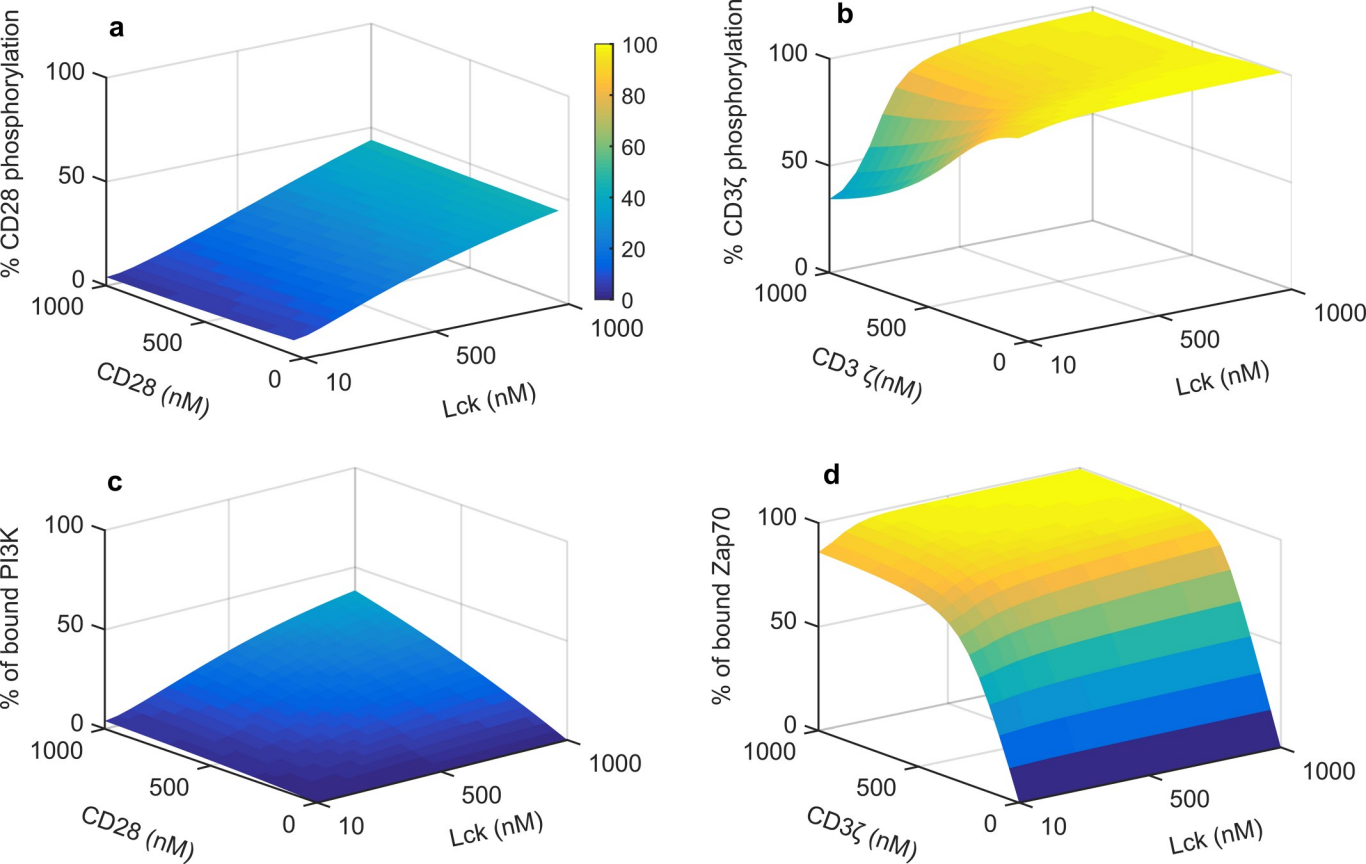
**Effect of 300 nM PD-1 and Shp2 on (a) CD28 phosphorylation, (b) CD3ζ phosphorylation, (c) PI3K recruitment and (d) Zap70 recruitment for a range concentrations of Lck and CD28 or CD3ζ**. Concentrations of other components are same as in Fig 3.

Now to assess the effect of PD-1, on the same variables we ran simulations with 300 nM PD-1 and 300nM Shp2 (Fig 5). Introduction of PD-1 leads to a dramatic reduction in CD28 phosphorylation and subsequent reduction in PI3K recruitment as compared to their response without PD-1 (Figure D in S1 File). Whereas CD3ζ phosphorylation is reduced only at low Lck concentration highlighting the differential sensitivity of CD28 and CD3ζ towards PD-1. Lck at higher concentrations could reduce the inhibitory effect of PD-1 on the phosphorylation of these receptors. Due to the less sensitive nature of CD3ζ to PD-1 the effect on association of downstream signaling molecule Zap70 is minimal (Fig 5D).

**Fig 6:**
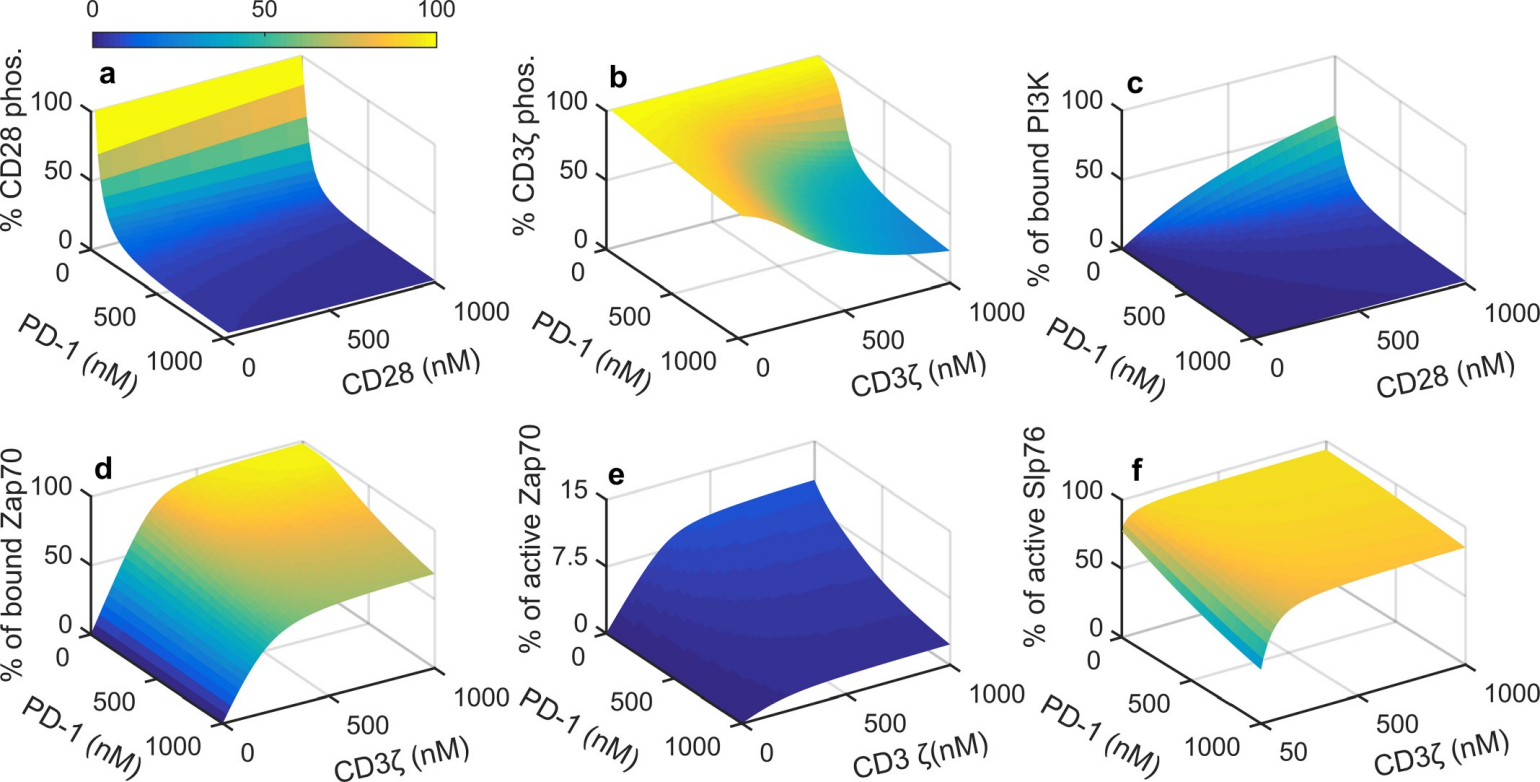
**Effect of PD-1 on (a) CD28 phosphorylation, (b) CD3ζ phosphorylation, (c) PI3K recruitment, (d)Zap70 recruitment, (e) activation of Zap70 (Zap70_a2_) and (f) activation of Slp76 (Slp76_a_) with 300 nM Shp2 and 100 nM Lck**.

In order to find out the effect of PD-1 for a range of its concentration, we performed a two dimensional scan of PD-1 and either CD28 or CD3ζ as appropriate (Fig 6) with 300 nM Shp2 and 100 nM Lck. PD-1 fully reverses the effect of Lck on CD28 even at its moderate concentration (~300 nM) resulting a complete loss of engaged PI3K (Fig 6A & 6C). Inhibition of CD3ζ phosphorylation happens at PD-1 concentrations greater than 500nM only when the CD3ζ concentration is also higher (Fig 6B). The maximal inhibition happens only at very high concentrations of PD-1 and CD3ζ. Recruitment of Zap70, its subsequent activation (Zap70_a2_) and activation of Slp76 are affected only at very high concentration of PD-1 (Fig 6D–6F). The effect of PD-1 on Slp76 is very weak as Slp76 is not a direct target of PD-1. Thus PD-1 causes a dual effect by a strong inhibition of CD28 pathway and a moderate inhibition of CD3ζ.

**Fig 7:**
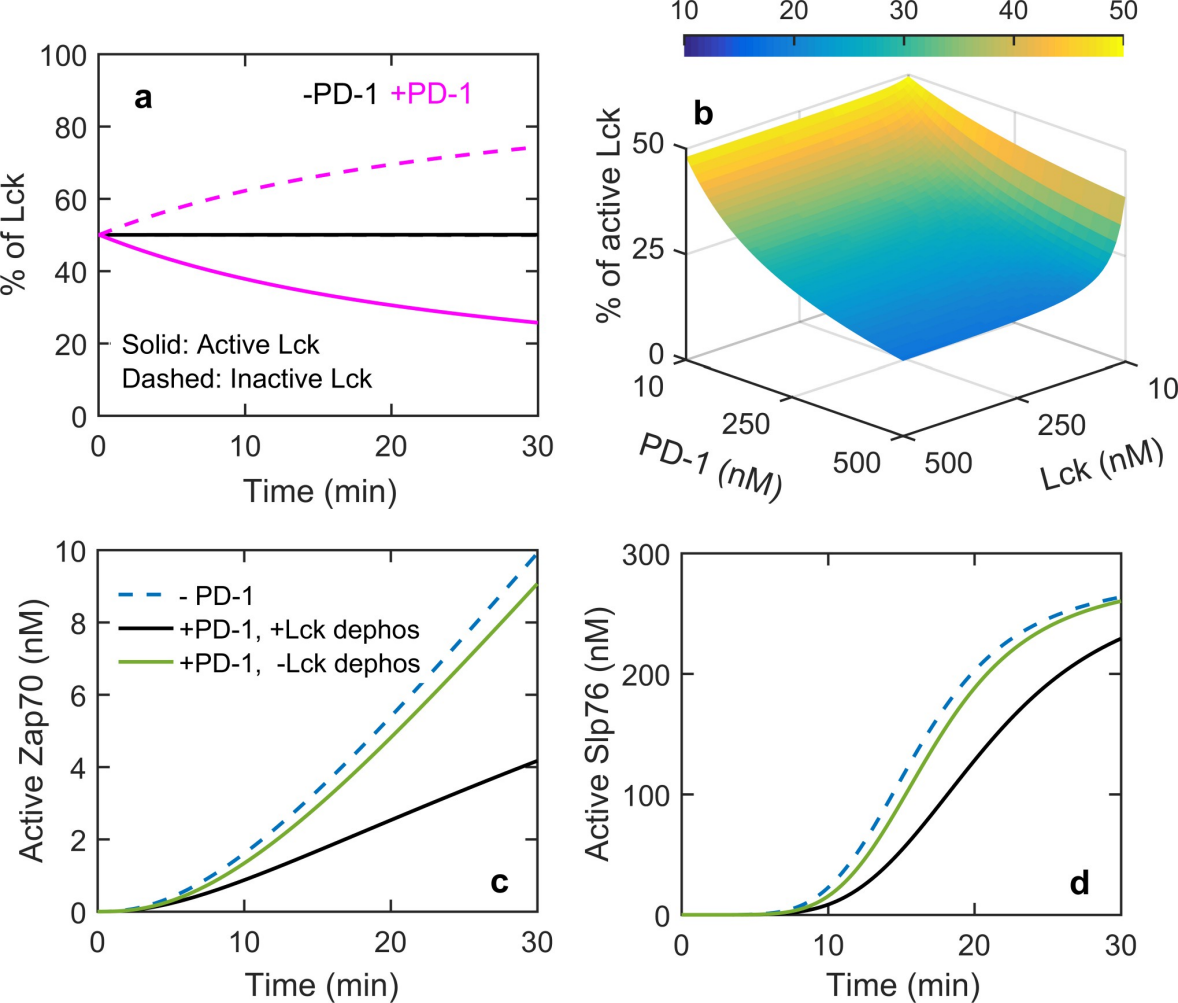
(a) Time course of active and inactive Lck with and without PD-1. (b) Percentage of active Lck at 30 minutes for different PD-1 and Lck concentrations. (c) Effect of Lck dephosphorylation on Zap70 Y493 phosphorylation and (d) Slp76 phosphorylation. The concentrations used for a-d are 100nM CD3ζ and Lck, 300 nM Zap70, Shp2, LAT, SLP76 and Gads. Concentration of PD-1 was 300 nM for (a) and 500 nM for (c) and (d).

PD-1 dose response simulations (and experiments) showed that PD-1 dephosphorylates both the activating (Y394) and inhibitory (Y505) tyrosine amino acid residues of Lck (Fig 3). Although, both the tyrosine amino acids are dephosphorylated to similar extent by PD-1 (similar profiles in the PD-1 dose response curve in Fig 3), due to the presence of a doubly phosphorylated species of Lck which retains catalytic activity, it is unclear if the dephosphorylation of Lck has a net activating or inhibitory effect. In the model Lck_yiya_ and Lck_ya_ are considered as active Lck forms capable of phosphorylating its substrates PD-1, ZAP70, CD28 and CD3ζ, and all other forms of Lck are considered as inactive. In the absence of PD-1, both the active and inactive Lck forms are present in equal proportion but in the presence of 300nM PD-1, proportion of inactive Lck increases over time (Fig 7A). A two dimensional variation of PD-1 and Lck shows (Fig 7B) that above 100 nM Lck concentration the proportion of active Lck decreases systematically with increasing PD-1 indicating its net inhibitory effect on Lck. At Lck concentrations below 100nM, the decrease in active Lck proportion with increasing PD-1 concentration is less and this is due to the requirement of Lck for PD-1 activation. Thus the model predicts that PD-1 imposes a net inhibitory effect on Lck and thereby causing an indirect inhibition to CD28 and CD3ζ signaling.

These observations raise the question, how significant is the inactivation of Lck in inhibiting the TCR and CD28 early signaling molecules. To explore this, we performed simulations where CP_1_ and CP_2_ (PD-1-Shp2 complexes) do not dephosphorylate Lck but dephosphorylate its usual target CD28 and CD3ζ. In the absence of Lck dephosphorylation by PD-1/Shp2 complexes, the inhibitory effect on Zap70 and Slp76 molecules are shown in Fig 7C and 7D. Although activation of CD3ζ and CD28 receptors were not greatly affected (not shown) but the activation of Zap70 and Slp76 are significantly affected without Lck dephosphorylation. Hence, the inhibitory effect of PD-1 on the downstream components such as the Zap70 and Slp76 are achieved indirectly via Lck dephosphorylation.

**Fig 8:**
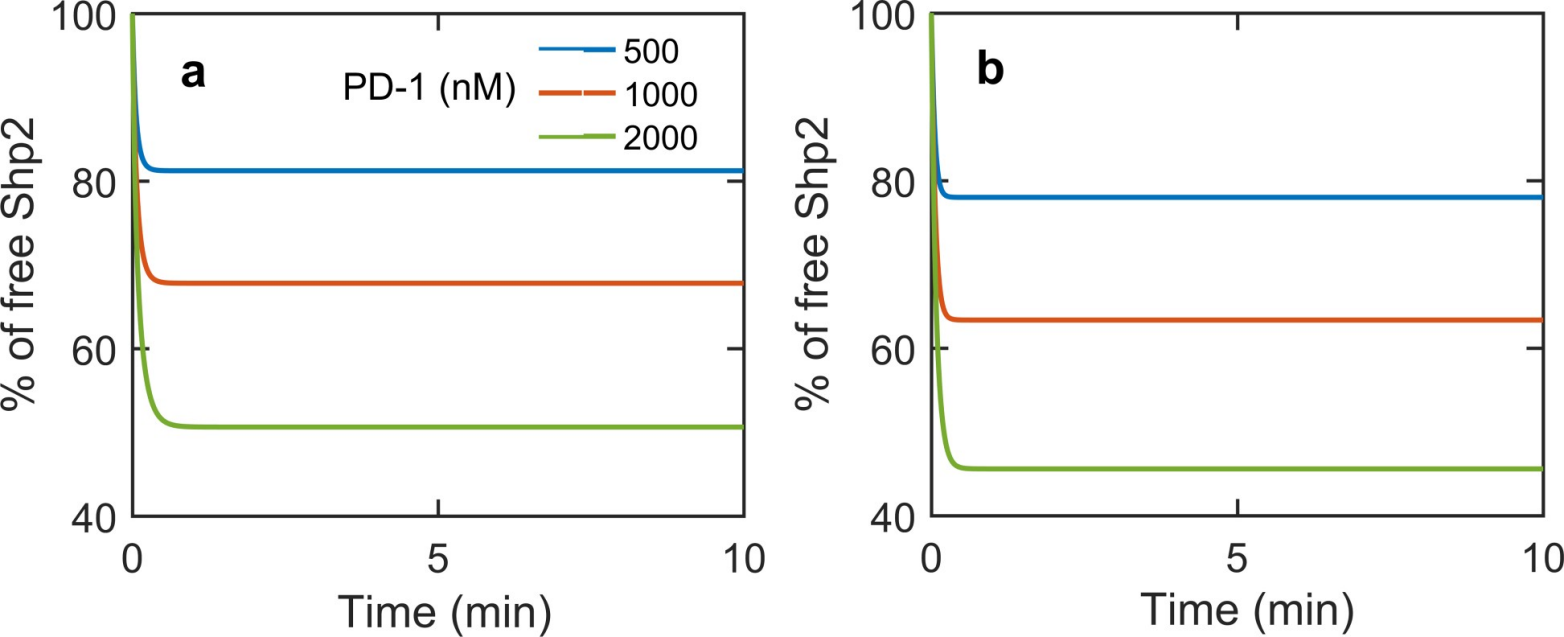
**Time course of free Shp2 in the absence of (a) Lck dephosphorylation and b) PD-1 dephosphorylation for different PD-1 concentrations with 100nM Lck and 300nM Shp2**.

We have shown that after rapid engagement Shp2 dissociates from the PD-1 at later time (Fig 2B). To test if self-dephosphorylation is solely responsible for this net dissociation or loss of Lck activity due to Lck dephosphorylation affects the net dissociation, we again carried out perturbation simulations of the model. As shown before without self-dephosphorylation and with Lck dephosphorylation there is no recovery of Shp2 (Fig 8B). However in the reverse condition also the model predicts no net dissociation of Shp2 from PD-1 (Fig 8A). These simulations show that both the self dephosphorylation and Lck dephosphorylation are collectively responsible for the observed net dissociation of Shp2 from PD-1. Previous simulations in Fig 7 show that the Lck is inactivated by this PD-1 induced dephosphorylation. Hence, the self dephosphorylation activity of CP_1_ and CP_2_ complexes, decrease their stability and the inactivation of Lck decreases the phosphorylation of PD-1 thus inhibiting the formation of these inhibitory complexes.

### Sensitivity analysis

Predictions and output of a mathematical model rely largely on parameter values. Hence, it is crucial to test the robustness of the model output to changes in parameters. Parameter sensitivity analysis is a technique extensively used for mathematical models to determine the fluctuations in the model output due to uncertainty in the parameters used. We used global parameter sensitivity analysis where all the chosen parameters are varied simultaneously to explore their collective effect on the dynamics of the system (see Materials and methods). Global sensitivity analysis offers advantage over the local parameter sensitivity analysis, as it explores the impact of the interaction between uncertainties in different parameter values and is considered more reliable [69]. Owing to the large number of optimized parameters, global sensitivity analysis was performed to test the robustness of Shp2 recruitment, PI3K recruitment and Slp76 activation in a ‘modular’ manner.

**Fig 9:**
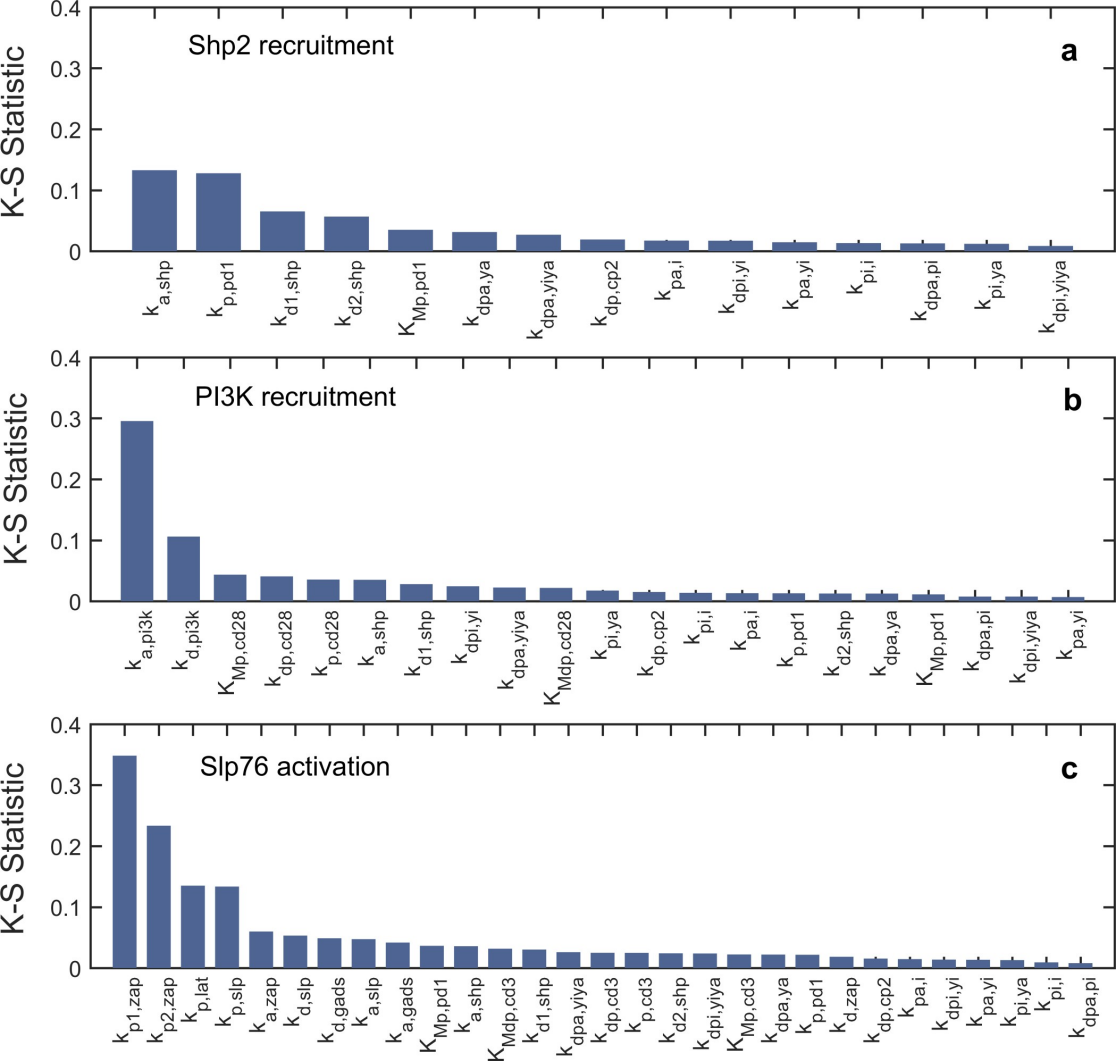
**Parameter sensitivity: Bar plot showing K-S Statistic measure of parameters tested for sensitivity of Shp2 recruitment (% of bound Shp2) (a), PI3K recruitment (% of bound PI3K) (b) and Slp76 activation (% of Slp76_a_) (c)**.

1) Shp2 recruitment by PD-1:

Dissimilarity measure, K-S statistic (see Materials and Methods) was calculated for parameters involved in the activation of PD-1 by phosphorylation, formation of CP_1_ and CP_2_ complexes and parameters for Lck auto-phosphorylation and dephosphorylation. This module includes 15 parameters (all other parameters were kept constant) and representative cumulative frequency distributions for most and least sensitive parameters were provided in Figure E in S1 File. The K-S statistic estimates for all of the 15 parameters are summarized in the bar plot given in Fig 9A. The results suggest that the percentage of Shp2 recruitment is influenced mainly by changes in the parameters k_p,pd1_ and k_a,shp_ which are the phosphorylation rate constant of PD-1 and association rate constant of Shp2 to phosphorylated PD-1. Their K-S statistic values are approximately 0.13. Shp2 recruitment is relatively robust to changes in the phosphorylation and dephosphorylation rate constants of Lck.

2) PI3K recruitment by phosphorylated CD28:

Sensitivities of PI3K recruitment to 21 different parameters were calculated keeping other parameters constant. Out of all the parameters tested, the parameter k_a,pi3k_, rate constant for association of PI3K to phosphorylated CD28 was the most influential, with a K-S statistic value of ~0.3, in affecting PI3K recruitment. Parameter k_d,pi3k_, dissociation rate constant of PI3K from CD28, had a K-S statistic value of 0.1. However, the K-S statistic for other parameters were less than 0.05, suggesting that PI3K recruitment is insensitive to a wider range of these parameter values around their optimized values (Fig 9B).

3) Slp76 activation:

Effect of uncertainties in 29 parameters on Slp76 activation was tested. The remaining 7 parameters involving CD28-PI3K module were kept constant. Among all the 29 parameters, K-S statistic for the parameters k_p1,zap_, k_p2,zap_, k_p,lat_ and k_p,slp_ are high (K-S statistic values are approximately 0.35, 0.2, 0.15 and 0.15 respectively), highlighting their relative importance in determining the Slp76 activation (Fig 9C). These parameters are the rate constants of phosphorylation of Zap70 by Lck, LAT and Slp76 by activated Zap70. Rest of the parameters had a K-S statistic value less than 0.05.

## Conclusion

We propose a mathematical model of PD-1 pathway that negatively regulates the TCR activation pathway. The model has been parametrized and benchmarked to quantitatively recapture kinetic and dose response experimental results from recently published paper by Hui et al[46]. Further it explored two dimensional dose responses of various signaling molecules for a wide concentration range of PD-1 and CD28 or CD3ζ. The model provides molecular insights into the inhibitory effect of PD-1 on several key regulators such as CD28, CD3ζ, PI3K, Zap70 and Slp76. A key finding of the model is that PD-1-Shp2 complex targets TCR pathway both directly and indirectly. On the direct path, it leads to dephosphorylation of CD28 and CD3ζ resulting a decrease in binding of PI3K and Zap70, respectively. On the indirect path, it dephosphorylates Lck leading to a net inhibitory effect on Lck and thereby it indirectly downregulates activation of Zap70, LAT, Slp76 in the TCR-CD3 pathway whose activation requires phosphorylated Lck. Therefore PD-1 causes a dual effect to the TCR activation pathway by downregulating CD28, CD3ζ directly and Zap70, LAT, Slp76 indirectly via Lck dephosphorylation. The model highlights the importance of Lck dephosphorylation by PD-1-Shp2 complex in downregulating the TCR pathway. Global parameter sensitivity analysis of the model finds crucial parameters in PD-1 mediated dephosphorylation of several key molecules. Higher parameter sensitivity of Lck mediated phosphorylation rate of Zap70 in the Slp76 activation pathway further suggests that the inhibition of Lck could have a relatively large impact on the early TCR signaling. Collectively, simulation results point out that the Lck could be a potential target employed by PD-1 pathway to inhibit the activation of several signaling components or alter several signaling pathways. Here we used *manual* approach in parameterizing our model to benchmark with the experimental data. We preferred the *manual* approach as our primary goal was to model the network of PD-1 pathway and not to estimate the parameter values of an established pathway. However one can use a parameter estimation tool box to optimize the model parameters.

Our proposed model is deterministic in nature considering all the cells in a population as identical in every aspect. Therefore the physiological basis of dose response curves with varying quantity/dose of signaling molecules such as Lck, PD-1, CD28 and CD3ζ in our model needs to be highlighted. A clonal population of cell exhibits large cell-to-cell variation in cellular content, shape, size and cell cycle phases due to intrinsic and extrinsic source of heterogeneity. The variations in expression of key signaling components, both in upstream and downstream pathways, ultimately lead to heterogeneous response across the population [70–74]. Although our model does not consider cell-to-cell variation of proteins, however the deterministic dose response curves provides an avenue to estimate the dynamic range of response and the critical amount of signal needed for the population to respond (IC_50_) for a heterogeneous population of cells. Therefore in a way our model sets the background for predicting the outcome of the system in presence of population heterogeneity.

A key feature in PD-1 pathway is the presence of a negative feedback loop involving PD-1 and Shp2 (Fig 1). In this motif activation of PD-1 leads to recruitment of Shp2 that ultimately deactivates PD-1 via dephosphorylation. A negative feedback loop is well known for its adaptation property that allows the system to respond to the external signal and reset back to its original state even in presence of persistent signal [75]. The resetting of the system is very crucial for T cell to avoid any unwanted over- or under-reaction of the immune system. Further for coherent and collective response of a population of T cell against pathogen the effect of inevitable noise must be minimized by the regulatory pathway. Previous experimental and computational studies have demonstrated that network architecture, in particular negative feedback loop and feed forward loop, play a crucial role in reducing molecular noise [76–81]. Therefore these network motifs in the PD-1 pathway may have been selected over evolution due to their role in reducing noise in addition to their usual deterministic properties.

Early TCR signaling has numerous potential implications. Recently, a study has shown a direct role of early TCR signaling in the activation of the enzyme pyruvate dehydrogenase kinase 1 (PDHK1). Consequently, mitochondrial import of pyruvate is inhibited and aerobic glycolysis is promoted [82]. Hence, early TCR signaling is important in regulating the aerobic glycolysis, which is characteristic of activated T cells. Model developed here could be employed to understand the inhibition of several early TCR signaling molecules due to PD-1 in the case of T cell activation or T cells exhausted due to chronic viral infections.

Experiments done on membrane based reconstitution system are considered physiologically more realistic when compared to traditional solution based experiments for signaling studies[62]. Although the model recaptures several of these observations, *in vivo* scenario could be considerably different from the network modeled here. Future improvements in the model could potentially result in a better prediction and reproduction of *in vivo* scenario. Although the impact of PD-1 on Lck activity was explored here, regulation of Lck activity is very complex due to the action of several other kinases and phosphatases such as Csk and CD45[83]. Model simulation could be made more realistic by considering the timescale of separation of TCR and PD-1 activation. This is because PD-1 expression itself is under the control of TCR signaling. In fact, recent studies have shown that the strength of TCR signaling influences PD-1 expression [84, 85]. Moreover, binding dynamics of receptors on the T cell surface to the ligands present on the surface of antigen presenting cells could significantly impact downstream activation. However, this model serves as a base to which such effects could be incorporated provided reliable experimental results or kinetic measurements are available.

Finally, the proposed model is based on a set of deterministic dynamical equations. Although our deterministic model recaptures many key experimental observations however being deterministic in nature it is not capable of explaining single cell data which requires stochastic modeling. It is established that in the early T cell receptor activation pathway population heterogeneity of key signaling proteins, such as Lck, Zap70, play a crucial role [70, 86]. However in this paper our main objective was to establish a mechanistic mathematical model of PD-1 pathway and stochastic calculations of the current model is definitely a scope of the future.

## Materials and methods

### Parameter sensitivity analysis

To implement global sensitivity analysis, we make use of multi parametric sensitivity analysis (MPSA) where multiple parameters are selected together for testing the sensitivity. Each parameter was picked from a uniform distribution with a range ±50% of the optimized parameter value used in the model (Table 2). We used Latin hyper cube sampling technique to create a sample of 20000 values for each parameter. Parameter samples were permuted randomly and a combination of parameters was taken as a parameter set, thus generating 20000 parameter sets. We calculated the parameter sensitivity on the time course data of various quantities. 11 different PD-1 concentration values were generated that include 0 nM and 10 logarithmically spaced between 1 and 1000 nM. In each PD-1 concentration, we calculated the sum squared deviation over 10 different time points with respect to the time course of optimized parameter. Finally we estimated the overall error by summing the squared deviation over all PD-1 concentrations. In order to determine acceptable parameter combination, we set a threshold by taking the average of the overall sum of squared error determined over 20000 parameter sets. Any parameter combination that results overall error below the threshold was considered as acceptable and otherwise it was considered unacceptable [87]. We calculated cumulative frequency distribution for individual parameter values from the acceptable and unacceptable parameter sets. See Figure E in S1 File for representative cumulative frequency distributions. To determine the sensitivity of a parameter we calculated Kolmogorov-Smirnov (K-S) statistic which quantifies the dissimilarity of two probability distributions by measuring the maximum perpendicular distance between their respective cumulative distribution functions. The more dissimilar the two distributions are the distance between the two distributions would be higher. Hence, higher the K-S statistic, higher is the sensitivity of that particular parameter[88]. Concentration of components used in the sensitivity analysis is close to physiological concentrations. Sensitivity analysis was performed separately for three different measures in the model–percentages of Shp2 recruitment, PI3K recruitment and active form of Slp76 pertaining to sensitivities of PD-1-Shp2 complex formation, early CD28 signaling and early TCR signaling, respectively.

## Supporting information

S1 File Contains all the supplementary figures and tables. (DOCX)
