# Supplementary material for: Mathematical modeling identifies Lck as a potential mediator for PD-1 induced inhibition of early TCR signaling
Source: PLoS One. 2018 Oct 24;13(10):e0206232. doi: 10.1371/journal.pone.0206232 (PMC6200280; doi:10.1371/journal.pone.0206232)
Supplement: S1 File — (DOCX) [file pone.0206232.s001.docx]

Supplementary Text to

**Mathematical modeling identifies Lck as a potential mediator for PD-1 induced inhibition of early TCR signaling**

Theinmozhi Arulraj^1^, Debashis Barik^2*^

^1^Centre for Systems Biology, School of Life Sciences, University of Hyderabad, Central University P.O., Hyderabad, 500046, Telangana, India

^2^School of Chemistry, University of Hyderabad, Central University P.O., Hyderabad, 500046, Telangana, India

^*^Corresponding author, Email address: [dbariksc@uohyd.ac.in](mailto:dbariksc@uohyd.ac.in)

**Table A:** Description of model variables.

|  | **Model Component** | **Description** |
| --- | --- | --- |
| 1 | Lck_yiya_ | Lck phosphorylated at both Y394 and Y505 (Active) |
| 2 | Lck_yi_ | Lck phosphorylated only at Y505 (Inactive) |
| 3 | Lck_ya_ | Lck phosphorylated only at Y394 (Active) |
| 4 | Lck_pi_ | Lck phosphorylated at both Y394 and Y505 (Inactive) |
| 5 | Lck_i_ | Unphosphorylated form of Lck (Inactive) |
| 6 | PD1 | Unphosphorylated form of PD-1 |
| 7 | PD1_p1_ | Monophosphorylated PD-1 |
| 8 | PD1_p2_ | Diphosphorylated form of PD-1 |
| 9 | Shp2 | Free Shp2 |
| 10 | CP_1_ | PD1_p1_/Shp2 complex |
| 11 | CP_2_ | PD1_p2_/Shp2 complex |
| 12 | CD28_i_ | Unphosphorylated CD28 |
| 13 | CD28_a_ | Phosphorylated CD28 |
| 14 | PI3K | Free PI3K |
| 15 | PI3K_b_ | CD28_a_/PI3K complex |
| 16 | CD3_i_ | Unphosphorylated CD3ζ |
| 17 | CD3_a_ | Phosphorylated CD3ζ |
| 18 | Zap70 | Free unphosphorylated Zap70 |
| 19 | Zap70_i_ | CD3_a_/Zap70 complex with unphosphorylated Zap70 |
| 20 | Zap70_a1_ | CD3_a_/Zap70 complex with Zap70 phosphorylated at Y315 |
| 21 | Zap70_a2_ | CD3_a_/Zap70 complex with Zap70 phosphorylated at Y315 and Y493(Active Zap70) |
| 22 | LAT_i_ | Unphosphorylated LAT |
| 23 | LAT_a_ | Phosphorylated LAT |
| 24 | Gads | Free Gads |
| 25 | Gads_a_ | LAT_a_/Gads complex |
| 26 | Slp76 | Free unphosphorylated Slp76 |
| 27 | Slp76_i_ | LAT_a_/Gads/Slp76 complex with unphosphorylated Slp76 |
| 28 | Slp76_a_ | LAT_a_/Gads/Slp76 complex with phosphorylated Slp76  (Active Slp76) |


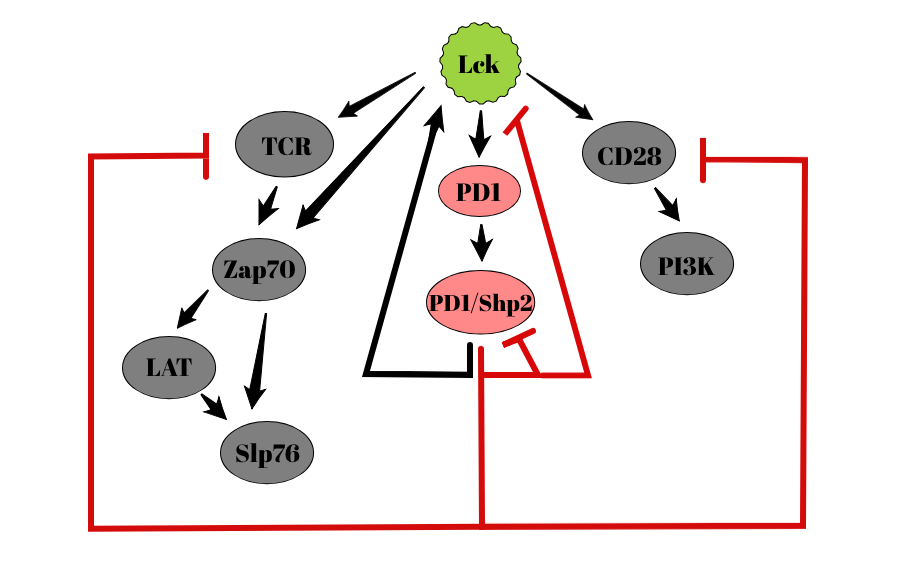


**Figure A:** Influence diagram of the model. Lck activates TCR and CD28 receptors which in turn activate downstream signaling molecules. Lck also activates the inhibitory receptor PD-1 resulting in the recruitment of Shp2. Shp2 bound PD-1, inactivates TCR and CD28 apart from deactivating itself. In addition, PD1/Shp2 complex has both inhibitory and activating effect on Lck via dephosphorylation.


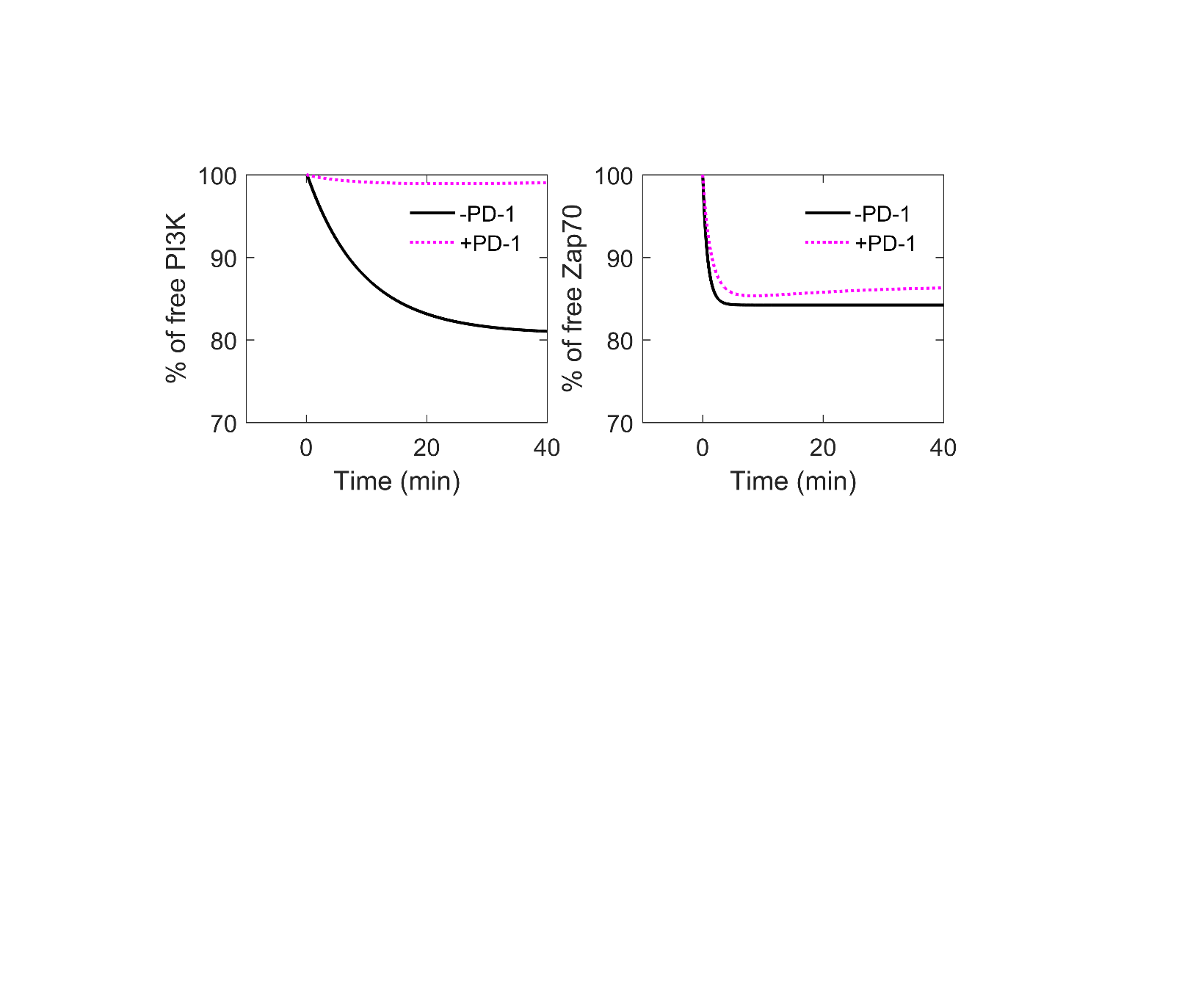


**Figure B:** Effect of PD-1 on the time course of recruitment of PI3K and Zap70 respectively. The concentrations used were 50 nM CD3ζ, 300 nM Zap70, 250 nM CD28, 500 nM PI3K, 300 nM Lck, 800 nM PD-1 and 500 nM Shp2.


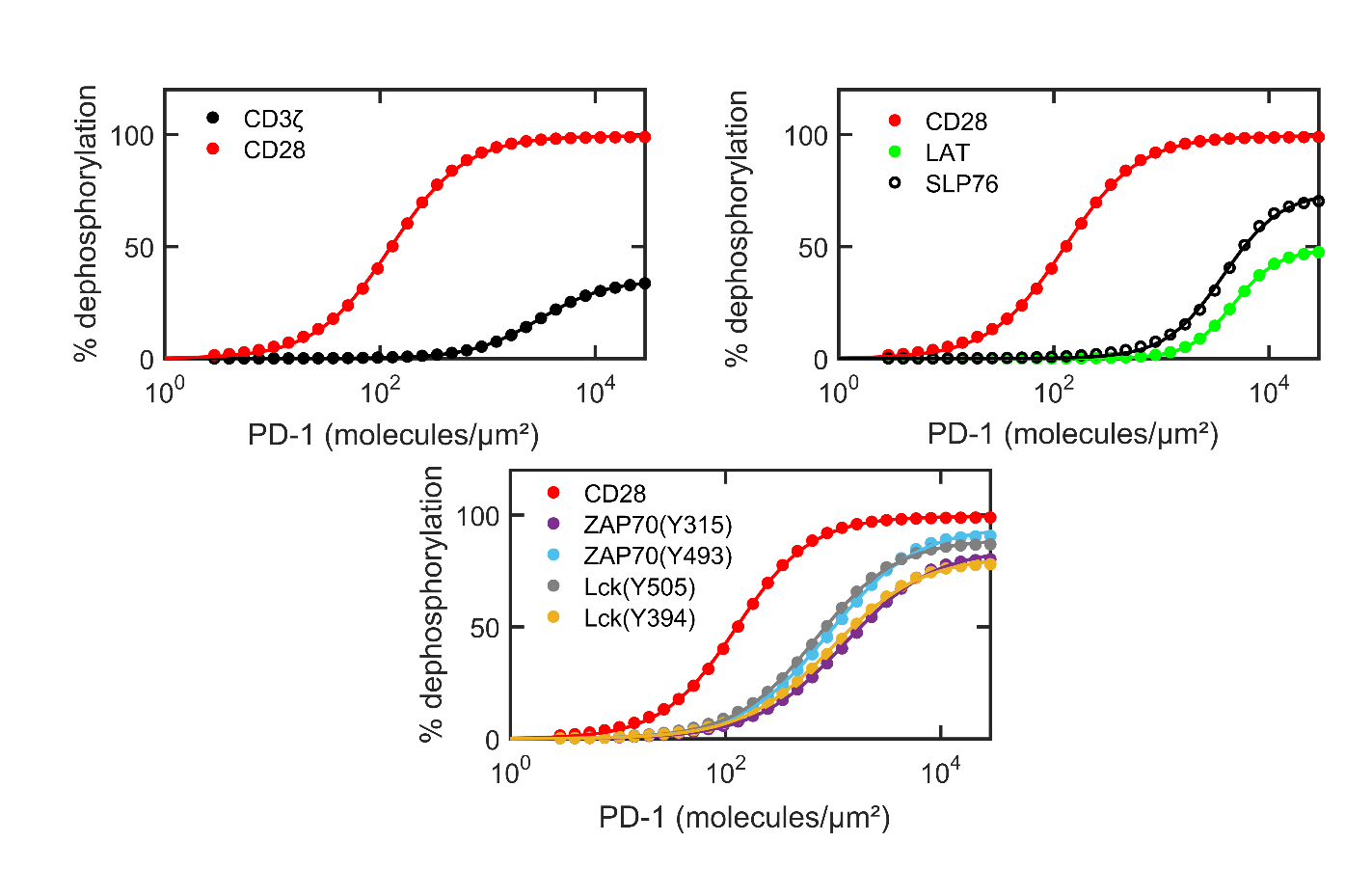


**Figure C:** Fitting the dose response curves with Hill function to determine IC_50_ and Hill coefficients of dose responses. Solid lines represent fitting of data using Hill function.


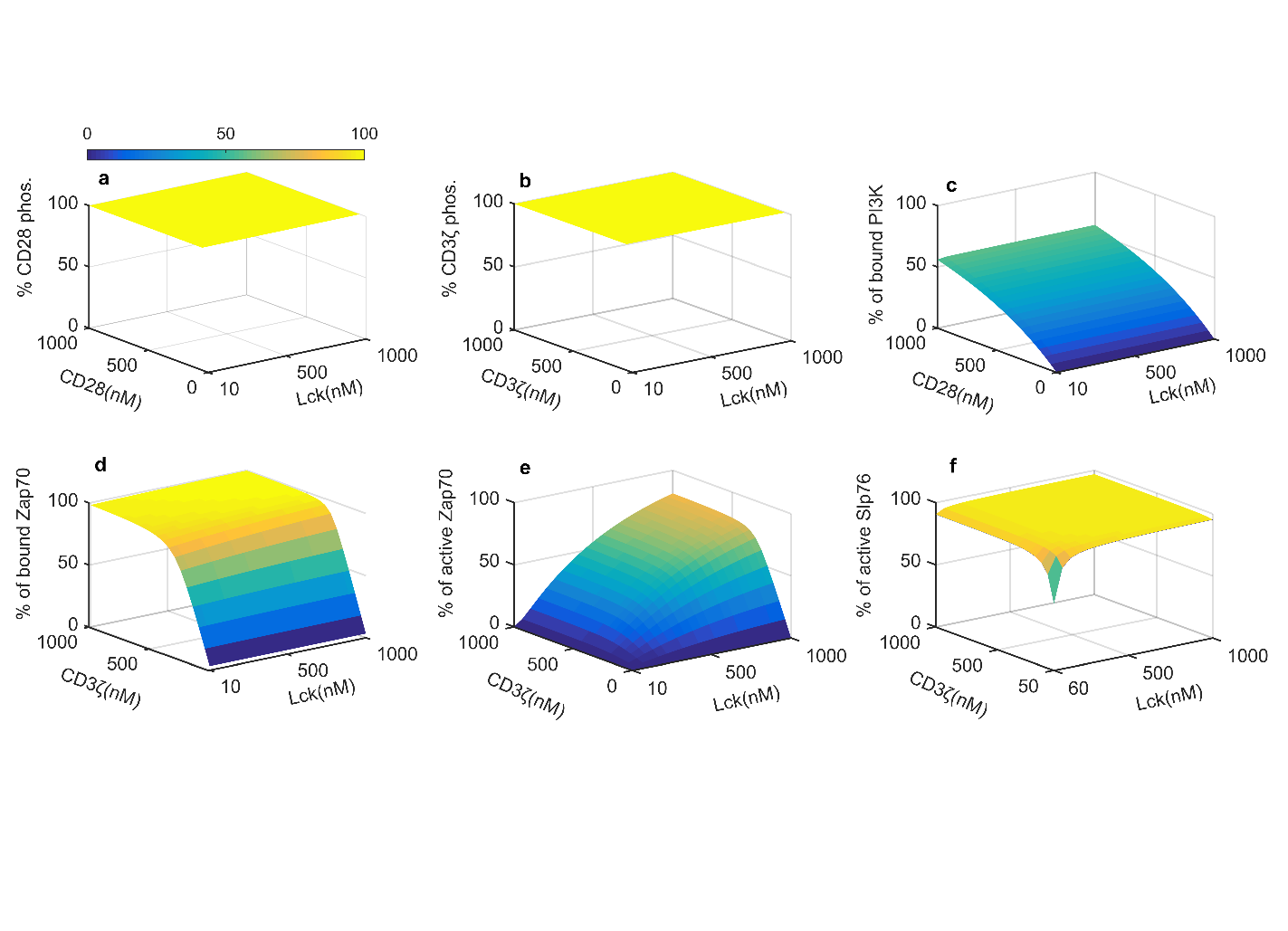


**Figure D:** Dependence of CD28 phosphorylation (a), CD3ζ phosphorylation (b), PI3K recruitment (c), Zap70 recruitment (d), percentage of active Zap70 (e) and percentage of active SLP76 (f) on Lck and CD28 or CD3ζ concentrations in the absence of PD-1 with 200nM PI3K, 300nM Zap70, LAT, Gads and SLP76.


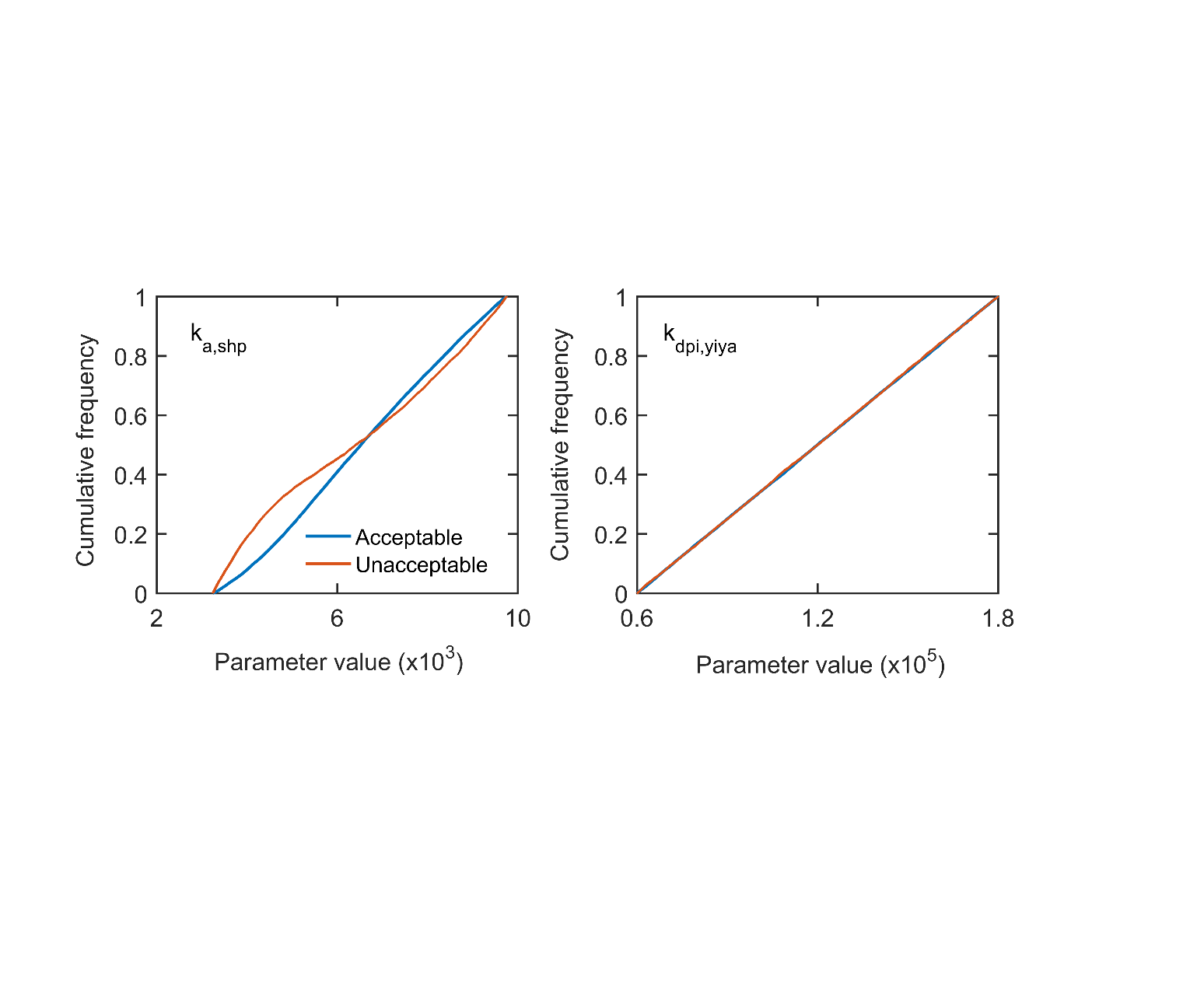


**Figure E:** Representative cumulative frequency distribution of accepted and unaccepted parameter values of k_a,shp_- rate constant for association of Shp2 to PD-1 and k_dpi,yiya_ – rate constant for dephosphorylation Y505 from Lck_yiya_.
